# *CACNA1A* loss-of-function affects neurogenesis in human iPSC-derived neural models

**DOI:** 10.1007/s00018-025-05740-7

**Published:** 2025-06-14

**Authors:** Ilaria Musante, Davide Cangelosi, Lorenzo Muzzi, Fanny Jaudon, Marco Di Duca, Sara Guerrisi, Francesca Antonini, Yeraldin Chiquinquira Castillo De Spelorzi, Lorenzo A. Cingolani, Federico Zara, Paolo Scudieri

**Affiliations:** 1 Medical Genetics Unit, IRCCS Istituto Giannina Gaslini, Genoa, Italy; 2 Clinical Bioinformatics Unit, IRCCS Istituto Giannina Gaslini, Genoa, Italy; 3 Department of Neurosciences, Rehabilitation, Ophthalmology, Genetics, Maternal and Child Health (DiNOGMI), University of Genoa, Genoa, Italy; 4 Department of Life Sciences, University of Trieste, Trieste, Italy; 5 Core Facilities for Omics Science, IRCCS Istituto Giannina Gaslini, Genoa, Italy; 6 Genomics Facility, Italian Institute of Technology (IIT), Genoa, Italy

**Keywords:** Neurodevelopmental diseases, In vitro models, Induced pluripotent stem cells, HD-MEA, ScRNA-seq

## Abstract

*CACNA1A* encodes the pore-forming α_1A_ subunit of the Ca_V_2.1 calcium channel, whose altered function is associated with various neurological disorders, including forms of ataxia, epilepsy, and migraine. In this study, we generated isogenic iPSC-derived neural cultures carrying *CACNA1A* loss-of-function mutations differently affecting Ca_V_2.1 splice isoforms. Morphological, molecular, and functional analyses revealed an essential role of *CACNA1A* in neurodevelopmental processes. We found that different *CACNA1A* loss-of-function mutations produce distinct neurodevelopmental deficits. The F1491S mutation, which is located in a constitutive domain of the channel and therefore causes a complete loss-of-function, impaired neural induction at very early stages, as demonstrated by changes in single-cell transcriptomic signatures of neural progenitors, and by defective polarization of neurons. By contrast, cells carrying the Y1854X mutation, which selectively impacts the synaptically-expressed Ca_V_2.1[EFa] isoform, behaved normally in terms of neural induction but showed altered neuronal network composition and lack of synchronized activity. Our findings reveal previously unrecognized roles of *CACNA1A* in the mechanisms underlying neural induction and neural network dynamics and highlight the differential contribution of the divergent variants Ca_V_2.1[EFa] and Ca_V_2.1[EFb] in the development of human neuronal cells.

**Supplementary Information:**

The online version contains supplementary material available at 10.1007/s00018-025-05740-7.

## Introduction

The gene *CACNA1A* encodes the pore-forming α_1 A_ subunit of the voltage-gated calcium channel Ca_V_2.1, also known as P/Q-type channel. This channel is abundantly expressed in various brain regions, with the cerebellum and the cerebral cortex showing the highest expression levels [1–3]. Within these regions, Ca_V_2.1 is found in different types of neurons and mostly localizes in presynaptic terminals, being critical for neurotransmitters release and synaptic plasticity [3–7]. The function of Ca_V_2.1 can be finely regulated through alternative splicing and variations in subunit composition depending on the developmental stage, tissue type, and specific cell context [3–5, 8–11]. In particular, *CACNA1A* undergoes extensive alternative splicing, resulting in several isoforms of the pore-forming α_1 A_ subunit, with differential domain organization, expression patterns, and channel properties [5, 9, 12]. For example, alternative splicing of the mutually exclusive exons 37a and 37b at the proximal C-terminus of the channel results in two divergent variants of an EF-hand-like domain, named EFa and EFb. These variants differ in their expression patterns, with Ca_V_2.1[EFb] predominating during early neurodevelopmental stages and Ca_V_2.1[EFa] being expressed only at later stages [13, 14]. Also, they exhibit different biophysical properties, with Ca_V_2.1[EFa], but not Ca_V_2.1[EFb], undergoing calcium-dependent facilitation, and they regulate synaptic transmission with different efficacy, with Ca_V_2.1[EFa] being the more effective variant [4, 9, 15, 16].

The relevance of Ca_V_2.1 in shaping brain development and function is demonstrated by the wide spectrum of episodic and progressive neurological disorders caused by mutations in the *CACNA1A* gene. Many heterozygous mutations have been associated with episodic ataxia type 2 (EA2), familial hemiplegic migraine type 1 (FHM1), and spinocerebellar ataxia type 6 (SCA6), as well as some forms of autism spectrum disorder [11, 17–22]. More recently, biallelic *CACNA1A* mutations have been found in patients with more severe clinical phenotypes, including early onset epileptic encephalopathy and progressive cerebral atrophy [23–25]. In general, *CACNA1A*-related disorders show large phenotypic variability, even within the same disease, possibly due to the variable effect of different types of mutations on Ca_V_2.1 channel function [26–36].

Heterologous expression of *CACNA1A* variants, coupled with cell imaging, biochemical, and electrophysiological studies, has provided insights into how certain mutations affect Ca_V_2.1 channel function [37–43]. Most mutations leading to EA2 cause loss-of-function due to either premature stop codons, resulting in the degradation of truncated protein products, or missense mutations, compromising protein maturation and trafficking or channel open probability and conductance [18, 37, 39, 41]. By contrast, FHM1 and some rare forms of EA2 are usually associated with gain-of-function mutations, resulting in enhanced open probability or conductance of Ca_V_2.1 [40, 44–46], whereas SCA6 is associated with abnormal CAG-repeat expansion within a C-terminal exon of *CACNA1A*, which may induce cytotoxicity of the resulting protein or dysregulated gene expression due to altered function of the α1 ACT protein [43, 47, 48].

Despite the growing understanding of the effects of different mutations on the properties of Ca_V_2.1 channels at the cellular level, the pathological mechanisms underlying *CACNA1A*-related neurological dysfunction are still to be elucidated. Furthermore, while studies based on knock out or genetically modified mouse models for *CACNA1A* have provided relevant insights [18, 49–55], the translation of such data to human disease remains challenging and underscores the need for suitable humanized models to unravel mechanisms underlying the *CACNA1A*-related pathological phenotypes.

To address this knowledge gap, we generated isogenic human iPSC-derived neural models carrying two loss-of-function mutations differentially affecting *CACNA1A* splice isoforms. Morphological, molecular, and functional analyses revealed an essential role of *CACNA1A* in neural induction, neuronal differentiation and maturation. Furthermore, our findings highlight the differential contributions of the Ca_V_2.1[EFa] and Ca_V_2.1[EFb] splice variants to human neuronal cells. The iPSC-derived neuronal models developed in this study will pave the way for future therapeutic testing for neurological disorders involving *CACNA1A*.

## Materials and methods

### HEK-293 cell culture and transfection

HEK-293 cells were cultured in Dulbecco’s modified Eagle medium (DMEM, Gibco) supplemented with 10% FBS, 2 mM glutamine, 100 U/mL penicillin, and 0.1 mg/mL streptomycin (complete culture medium) and were maintained in a 5% CO2 humidified incubator at 37 °C. Transfection was performed in 60%–70% confluent cultures seeded in 60 mm dishes at 80,000 cells/dish in complete culture medium the previous day. Cells were transfected with 12 µg DNA/dish using the Ca^2+^ phosphate method [56], and used 48 h post-transfection.

### Biotinylation assay and Western Blot

After three washes in ice-cold PBS, transfected HEK-293 cells were incubated with 2 mM of EZ-Link Sulfo-NHS-LC-Biotin (A39257, Thermo Fisher Scientific) diluted in PBS for 30 min on ice with gentle rocking. Free biotin was quenched three times with PBS containing 100 mM glycine. Cells were then lysed in 400 μl of RIPA buffer (50 mM Tris–HCl pH7.4, 150 mM NaCl, 2 mM EDTA, 1% NP-40, 0,1% SDS) supplemented with protease and phosphatase inhibitors (complete EDTA-free protease inhibitors [1187358001, Roche]; serine/threonine and tyrosine phosphatase inhibitors [P0044 and P5726, Sigma Aldrich]). Total lysates were centrifuged at 15,000 rpm for 15 min at 4 °C and 300 µl of the resulting supernatant was incubated with 40 µl of NeutrAvidin-conjugated agarose beads (29,200, Thermo Fisher Scientific) overnight at 4 °C. The remaining supernatant was kept as input. The depleted lysate (intracellular fraction) was removed, and the beads were washed four times with 500 µl of RIPA buffer before elution of the precipitated proteins (biotinylated extracellular fraction) with 2X gel loading buffer (10% SDS, 50% glycerol, 300 mM Tris HCl pH 6.8, 0,005% Bromophenol blue, 10% β-mercaptoethanol) for 10 min at 70 °C.

Proteins were separated by SDS-PAGE using 5% acrylamide gels and transferred on polyvinylidene fluoride (PVDF) membranes. After incubation with primary rabbit anti-Ca_V_2.1 (1:500; 152,203, Synaptic Systems) or rabbit anti-β-tubulin III (1:1,000; T2200, Sigma Aldrich) antibodies, membranes were incubated with secondary HRP-conjugated goat anti-rabbit antibody (1: 5,000; 31,460, Thermo Fisher Scientific) and immunocomplexes were detected with the chemiluminescent substrate (RPN2106, ECL Prime Western Blotting System, GE Healthcare). Chemiluminescent signals were acquired using a ChemiDoc imaging system (Biorad) and quantified using ImageJ (http://rsb.info.nih.gov/ij).

### Generation of isogenic iPSC lines carrying *CACNA1A* mutations

For the generation of iPSC lines carrying *CACNA1A* variants we used a control iPSC line purchased from Applied StemCell, catalog number: ASE-9211. Control iPSCs were maintained in vitronectin-coated plates and Essential 8 Flex medium (A2858501, Thermo Fisher Scientific) and passaged 1:5 twice a week by dissociation with Versene. Donor information: age: neonatal; sex: male; ethnicity: African American; clinical information: normal/healthy.

Isogenic iPSC lines carrying either F1491S (Chr19:13,257,474 T > C) or Y1854X (Chr19:13,228,768 C > G) were generated by Applied StemCell CRISPR/Cas9-mediated gene editing service. Sequences of gRNAs and donors used for gene editing are reported in the Resource table (Supplementary information file). After iPSC clones’ selection and expansion, the presence of the desired mutations was confirmed by Sanger sequencing, while array-CGH with the SurePrint G3 Human CGH Microarray Kit 180 K (G4449 A, Agilent) detected no chromosomal rearrangements. Two independent clones were obtained for the F1491S mutation, while only one clone was obtained for the Y1854X mutation, probably due to a low-scoring CRISPR targeting efficiency of exon 37a sequence.

### Generation of iPSC-derived NPCs

iPSC-derived NPCs were generated by dual inhibition of SMAD signaling using the STEMdiff Neural System (STEMCELL Technologies) according to manufacturer’s instructions. Briefly, iPSC colonies were detached by incubation with StemPro™ Accutase™ Cell Dissociation Reagent (A1110501, Thermo Fisher Scientific) for 5 min and cells were collected by pipetting with DMEM/F12 medium (11,330,057, Thermo Fisher Scientific) to make a single cell suspension. The cells were then centrifuged at 300 g for 5 min and resuspended in STEMdiff Neural Induction Medium + SMADi (08581, STEMCELL Technologies) supplemented with RevitaCell (A2644501, Thermo Fisher Scientific). 3 × 10^6^ cells were seeded in each well of an AggreWell 800 plate (34,811, STEMCELL Technologies) in a total volume of 2 ml. The plate was centrifuged at 100 × g for 3 min to capture the cells in the microwells, and ¾ of the medium was changed daily for 5 days. Over this time, the iPSCs collected at the bottom of the well aggregated to form embryoid bodies (EBs). They were collected and plated into 6-well plates coated with Geltrex™ (A1413302, Thermo Fisher Scientific) for additional 7 days to form neural rosettes. Then, neural rosettes were detached using STEMdiff Neural Rosette Selection Reagent (05832, STEMCELL Technologies) and transferred to one well of a 6-well plate coated with Geltrex. NPCs grown out of neural rosettes were cultured for 5–7 days. NPCs were detached with accutase to make a single cells suspension and transferred to a 6-well plate in STEMdiff Neural Progenitor medium (05833, STEMCELL Technologies), at a density of 1 × 10^6^ per well as NPCs passage 1. NPCs were expanded and fed every other day with Neural Progenitor medium.

### Generation of iPSC-derived neurons

Isogenic control and mutated NPCs were plated in STEMdiff Neural Progenitor medium on Geltrex-coated supports (chamber-slides for immunofluorescence analysis or 24-well plates for RNA extraction) at a relatively low cell density (2 × 10^4^ cells/cm^2^) to allow the morphological analysis of developing neurons during the initial weeks of differentiation. After 24 h, the medium was substituted with Neurobasal Medium (21,103,049, Thermo Fisher Scientific) supplemented with GlutaMax Supplement (1:100), B-27 supplement (1:50), BDNF (10 ng/ml), GDNF (10 ng/ml), retinoic acid (1 μM), ascorbic acid (200 μM) and CulturOne supplement (1:100). Half of the medium was replaced twice a week during continuous culturing. From 5 DIV (DIV: days of differentiation from NPCs seeding), Neurobasal Medium and B-27 supplement were substituted by Neurobasal Plus Medium (A3582901, Thermo Fisher Scientific) and B-27 Plus supplement (A3582801, Thermo Fisher Scientific). Neuronal cultures were then fixed at different time points between 7 and 42 DIV for morphological characterization by immunofluorescence detection of selected markers.

For electrophysiological recordings on HD-MEA and single-cell transcriptomic analysis of control and Y1854X cells, the seeding protocol was slightly changed in order to avoid the formation of cell clumps over long-term culturing. Accordingly, NPCs were cultured at confluency for 48 h in Geltrex-coated flasks and Neurobasal Medium supplemented with GlutaMax Supplement (1:100), B-27 supplement (1:50), BDNF (10 ng/ml), GDNF (10 ng/ml), retinoic acid (1 μM). Cells were then harvested and plated at 1.4 × 10^5^ cells/cm^2^ on Poly-L-Ornithine/Laminin-coated supports (Accura HD-MEA, 3Brain AG, for electrophysiological recordings or 12-well plates for single-cell transcriptomics) in Neurobasal Medium supplemented with GlutaMax Supplement (1:100), B-27 supplement (1:50), BDNF (10 ng/ml), GDNF (10 ng/ml), retinoic acid (1 μM), ascorbic acid (200 μM) and CulturOne supplement (1:100). Media exchange schedules (half-medium change twice a week) and formulations (with Neurobasal Plus Medium and B-27 Plus supplement from the fifth day onwards) were instead unchanged. After DIV 35 CulturOne supplement and ascorbic acid were removed from media formulation.

### Real time qPCR

Quantification of total and isoform-specific *CACNA1A* mRNA was done by real time qPCR. RNA extraction was performed using RNeasy Mini Kit (74,104, Qiagen), according to the manufacturer’s protocol. The RNA concentration was measured with NanoDrop 1000 spectrophotometer (Thermo Scientific). 250 ng of total RNA was used for cDNA synthetis with iScript™ cDNA Synthesis Kit (1,708,891, Biorad). The sequences of forward and reverse primers used are listed in the Key Resources Table. PCR was performed in a CFX-96 real-time thermal cycler (Bio-Rad) using SsoFast EvaGreen Supermix (1,725,201, Bio-Rad).

### Immunofluorescence and cell imaging

#### Isogenic iPSC lines

Quality controls of isogenic control and mutated iPSC lines included the detection and quantification of classical undifferentiated state markers by immunofluorescence. For this purpose, iPSC lines were seeded on vitronectin-coated 8 Well Chamber – slides (80,841, Ibidi) in Essential 8 Flex medium (A2858501, Thermo Fisher Scientific). After 48 h, iPSCs were washed in PBS and fixed by adding 200 μl of 10% neutral buffered formalin (05-01005Q, Bio-Optica) for 5 min at room temperature. After 3 washings in PBS, cells were permeabilized with Triton X-100 0.3% in PBS for 5 min, blocked with 1% BSA in PBS for 2 h, and then incubated overnight at 4 °C with 200 μl of a solution of primary antibodies diluted in PBS containing 1% BSA. The following primary antibodies and dilutions were used: rabbit anti-OCT4 antibody (703,927, Thermo Fisher Scientific) at 1:500, mouse anti-SSEA4 antibody (MA1-021, Thermo Fisher Scientific) at 1:500, mouse anti-TRA-1–60 antibody (MA1-023, ThermoFisher Scientific) at 1:250, rat anti-SOX2 antibody (14–9811-82, Thermo Fisher Scientific) at 1:250. Following incubation with primary antibody, cells were rinsed 3 times in PBS and incubated with 200 μl of a solution of secondary Alexa Fluor–conjugated antibodies (Thermo Fisher Scientific) diluted 1:200 in PBS containing 1% BSA for 1 h in the dark. After further 3 washes in PBS, the chambers were removed, and the slides mounted with Fluoroshield with DAPI to stain cell nuclei.

Image acquisition was performed using a laser scanning confocal microscope TCS SP8 (Leica Microsystems) and an Eclipse TiE automated microscope (Nikon) to acquire high-resolution representative images and for quantitative analysis, respectively. Image analysis was performed using the General Analysis package in NIS Elements AR software. After applying background subtraction and autocontrast with fixed parameters, image segmentation was performed with threshold and morpho-separate object tools to detect and count nuclei (stained with DAPI). Combined analysis with “AND” operation between nuclei mask and nuclear markers’ masks was performed to quantify the percentage of OCT4- and SOX2-positive cells. Such combined analysis was preceded by dilate (1.5 μm) function on the nuclei binary mask to define the cell bodies and count those positive for the surface antigens SSEA-4 and TRA-1–60. 2000–6000 cells were analyzed for each sample and marker at the cell passages preceding the neural induction.

#### NPCs

NPC lines were tested for the expression of classical neural progenitor markers at passage 3. Cells were seeded on Geltrex-coated 8 Well Chamber – slides (Ibidi) and fixed after 48 h following the same protocol used for the iPSCs (see above). The following primary antibodies and dilutions were used: mouse anti-PAX6 antibody (MA1-109, Thermo Fisher Scientific) at 1:200, rabbit anti-SOX1 antibody (MA5-32,447, Thermo Fisher Scientific) at 1:200, rabbit anti-SOX2 antibody (PA1-094X, Thermo Fisher Scientific) at 1:500, mouse anti-Nestin antibody (MA1-5840, ThermoFisher Scientific) at 1:500.

Image acquisition and analysis were performed as described for the iPSC lines to quantify the percentage of NPC expressing neural markers. In addition, after image segmentation, the mean fluorescence intensity was extracted for each marker at single-cell level. 2000–6000 cells were analyzed for each sample and marker during 3 cell passages (P2-P3-P4) before terminal differentiation.

#### Neurons

Neurons were cultured on Geltrex-coated µ-Slide 8 Well (80,806, Ibidi) and then fixed and stained at different time points following the same protocol used for iPSCs and NPCs. The following primary antibodies and dilutions were used: guinea pig anti-beta3-Tubulin antibody (302 304, Synaptic Systems) at 1:200, mouse anti-NeuN antibody (MAB377, Sigma Aldrich) at 1:100, guinea pig anti-MAP2 antibody (188 004, Synaptic Systems) at 1:500, mouse anti-Neurofilament Marker SMI-312 antibody (837,904, BioLegend) at 1:100, mouse anti-GFAP antibody (173 011, Synaptic Systems) at 1:500, rabbit anti-Ki67 antibody (ab15580, Abcam) at 1:100.

Image acquisition and analysis were performed as described for the iPSC and NPC lines. The percentage of NeuN-positive cells was determined as done for the nuclear markers in the iPSCs experiments. For neurite outgrowth measurement, the dilate function (by 2.5 μm) was applied on the nuclei binary mask to define cell bodies. Then, neurite outgrowth was measured in terms of MAP2-positive area after cell-body (soma) subtraction and normalized on the total number of cells in the image. Axon specification was quantified by SMI-312-positive area after subtraction of soma and neurites area. 100–1000 cells were analyzed for each sample, marker, and biological replicate.

### Wound healing and proliferation assays

NPCs were seeded into a Geltrex-coated µ-Slide 8 Well (80,806, Ibidi), 2,5 × 10^5^ cells/well, and grown to confluency. A scratch wound was then made using a sterile 200-μl micropipette tip. Wound closure was evaluated at the indicated times by automated brightfield images acquisition using an Eclipse TiE automated Nikon microscope. The wound gap area was quantified with ImageJ software (NIH, Bethesda, MD, USA) by applying Find Edges and Sharpen processing tools and Analyze Particles tool.

NPCs proliferation was evaluated by adding 10 μM BrdU (ab142567, Abcam) in the culture medium during the 24 h following the scratch. At 24 h, NPCs were washed with PBS, fixed with 10% neutral buffered formalin for 5 min, and treated with 2 M HCl in 0.1% PBS-Tween for 30 min at room temperature. After 3 washings with PBS, cells were permeabilized with 0.3% Triton X-100 for 5 min and blocked with 1% BSA in PBS for 1 h. Then, cells were incubated overnight at 4 °C with a rat anti-BrdU antibody (ab6326, Abcam) diluted 1:250 in PBS containing 1% BSA. After further 3 washings in PBS, cells were incubated with a PBS solution containing 1% BSA, an Alexa flour-488-conjugated secondary antibody (A-21208, ThermoFisher) diluted 1:500, and 1 μg/ml Hoechst 33,342 (to label cell nuclei) for 1 h at room temperature in the dark. After washings, NPCs were imaged with an Eclipse TiE automated Nikon microscope and the percentage of BrdU-positive cells calculated using the General Analysis tool in Nikon NIS Elements AR software.

### Analysis of apoptosis

Apoptosis evaluation was performed on NPC and neurons at 10 DIV cultured as done for immunofluorescence analysis.

#### Apoptosis detection by flow cytometry

Control and mutant NPCs or neurons were gently detached with accutase and stained with Annexin-V and Propidium Iodide (PI) according to manufacturer instructions (Annexin V FITC Apoptosis Kit, catalog number: BMS500 F1-100, Thermo Fisher Scientific). After washing, cells suspensions were immediately analyzed by flow cytometry using the Beckman Coulter CytoFLEX SRT. Forward scatter (FSC) and side scatter (SSC) were used to exclude debris and assess cell viability. Annexin-V FITC fluorescence was detected in the FL1 channel (530/30 nm), while PI fluorescence was detected in the FL4 channel (670/30 nm). Data acquisition was performed for a minimum of 10,000 events, and analysis was conducted using Kaluza software. Results were expressed as the percentage of apoptotic cells (Annexin-V-positive/PI-negative) relative to the total cell population.

#### Apoptosis detection by cleaved-caspase-3 immunofluorescence

Control and mutant neurons were cultured on Geltrex-coated µ-Slide 8 Well (80,806, Ibidi) for 10 DIV and then fixed and stained following the same protocol described above. The following primary antibodies and dilutions were used: mouse anti-PAX6 antibody (MA1-109, Thermo Fisher Scientific) at 1:200 as a marker of early neurons and rabbit anti-Caspase-3 active form antibody (AF835, R&D System) at 1:500 as marker of apoptotic cells.

Image acquisition and analysis were performed as described above. The percentage of apoptotic cells was determined as done for the other nuclear markers quantified in iPSCs, NPCs, and neurons.

### Single-cell transcriptomics

#### Preparation of single-cell samples, libraries construction and sequencing

iPSC-derived NPCs or neurons were washed in PBS and detached as single cells by 5 min incubation with accutase. Single cells were suspended in PBS w/o Ca^2+^/Mg^2+^ containing 0.04% BSA, filtered using a 70 μm cell strainer Flowmi™ (734–2709, VWR), and counted with LUNA-II Automated Cell Counter (Logos Biosystems). Cell suspensions were loaded onto the Chromium Next GEM Chip G (10 × Genomics) and run on the Chromium Controller (10 × Genomics) to generate single-cell gel bead emulsion, according to the manufacturer’s protocol (User Guide—Rev E). The Chromium Next GEM Single Cell 3’ Kit v3.1 and the Chromium™ Next GEM Chip G Single Cell Kit (10 × Genomics) were used to generate cDNA and the final libraries. The cDNA quality was assessed using high-sensitivity D5000 screen tape on TapeStation system 4150 (Agilent Technologies). Quality of libraries was assessed by using high sensitivity D1000 screen tape (Agilent Technologies). Finally, the libraries were sequenced on Novaseq6000 sequencer using an S1 flow cell 100 cycles kit (20,028,319, Illumina), in pair-end mode (read1: 28 bp; read2: 90 bp), and with a depth of sequencing of 50,000 reads/cell.

#### scRNAseq data pre-processing and visualization

Raw sequencing data (FASTQ files) were processed by Cell Ranger [57] version 7.2.0 to obtain feature-barcode matrices. GRCh38-2020-A was used as reference transcriptome for aligning sequencing reads in FASTQ files. Estimated Number of Cells, Mean Reads per Cell, and Median Genes per Cell from Cell Ranger count pipeline report were used as metrics to evaluate data quality control. scRNAseq data files were imported, normalized, scaled and clustered using Seurat R package [58] version 5.1.0. Cell heterogeneity was visualized using uniform manifold approximation and projection (UMAP) [59].

#### Identification of marker genes in scRNAseq cell clusters

The function ‘FindAllMarkers’ with parameters “only.pos = TRUE, min.pct = 0.25” in the R package Seurat [58] was used to identify marker genes. Top 10 marker genes of each cell cluster were selected and visualized by the function ‘DoHeatmap’.

#### scRNAseq cell annotation and gene regulatory network inference

Cell identity annotation was performed using ScType method [60]. ‘Brain’ cell markers were used for cell annotation. ScType score lower than a quarter of the number of cells in a cluster or a negative ScType score were considered low-confidence cell type annotations and were assigned to “unknown” cell types [60].

To gain insight into the mechanisms driving cellular heterogeneity, the activity of the gene regulatory networks in each cell was evaluated using single-cell regulatory network inference and clustering (SCENIC) [61]. Nextflow pipeline using Docker profile with parameters “TFs: hs_hgnc_curated_tfs.txt, motifs: motifs-v10nr_clust-nr.hgnc-m0.001-o0.0.tbl, db: *feather, hr_min_genes: 1”. genome-ranking.feather and hg38\_refseq-r80_10kb_up_and_down_tss.mc9nr.genes_vs_motifs.rankings.feather were used as precomputed databases for co-expression modules and motif enrichments. The function ‘calcRSS’ was used to calculate the regulon specificity score. The function ‘binarizeAUC’ was used to binarize regulon activity. ComplexHeatmap R package was used to visualize binarized regulon activities.

### Electrophysiological analysis by HD-MEA recording

Extracellular recording of neuronal networks activity was done using Accura HD-MEAs (3Brain AG), high-density multi-electrode arrays presenting an active area of 3.8 mm × 3.8 mm with 4096 C-MOS electrodes, and the BioCAM DupleX (3Brain AG) instrument. Five minutes of full frame raw electrophysiological signals were recorded with BrainWave 5 software once every week between 21 and 60 DIV in incubator-like conditions (37 °C, 5% CO_2_). Data was sampled at 20 kHz. The array-wide firing rate was evaluated for each culture to show an overview of the total activity of the culture. Whole recordings were divided in 1 s bins and for each bin the total number of detected spikes were evaluated and divided by the bin lengths.

For spike detection, we used the Precise-Timing Spike Detection (PTSD) implemented in BrainWave 5. A threshold of 8 times the standard deviation factor of the noise (THsd) was used, peak-lifetime period of 1 ms, refractory period of 1 ms and the timestamp was assigned to the higher peak of the AP. The mean firing rate was evaluated for each electrode as the ratio between all the detected spikes and the recording time; only active electrodes exhibiting a mean firing rate > 0,1 spikes/second were considered for the analysis. The mean bursting rate was evaluated as the sum of all the detected bursts occurred in an active channel (a burst was defined as a series of 3 consecutive spikes firing no more than 100 ms apart from each other) divided by the recording time; only bursting electrodes having a mean bursting rate > 0,1 bursts/minute were considered. The mean burst duration was evaluated by averaging all the burst duration detected in the whole device. The percentage of random spikes was evaluated as the ratio between the total number of non-burst spikes and the total number of spikes. Similarly, the percentage of bursting channels (electrodes) was evaluated as the ratio between bursting electrodes and active electrodes. Synchronous events occurring within the networks, defined as network bursts, were detected as previously reported [62]. All data analysis after the spike detection was carried out using custom Matlab scripts.

### Quantification and statistical analysis

Data are shown as representative images and quantitative graphs reporting single data points and/or mean ± SEM obtained from different biological replicates. The precise number of biological replicates for each experiment are indicated in figure legends. To assess significant differences between groups of data, we used the Kolmogorov–Smirnov test to assess normal distribution, and then used 2-tailed Student’s t test to compare each mutant lines with the isogenic control, or 1-way ANOVA (followed by Tukey’s post hoc test), for more than 2 groups.

## Results

### Isogenic iPSCs carrying *CACNA1A* loss-of-function mutations

**Fig. 1:**
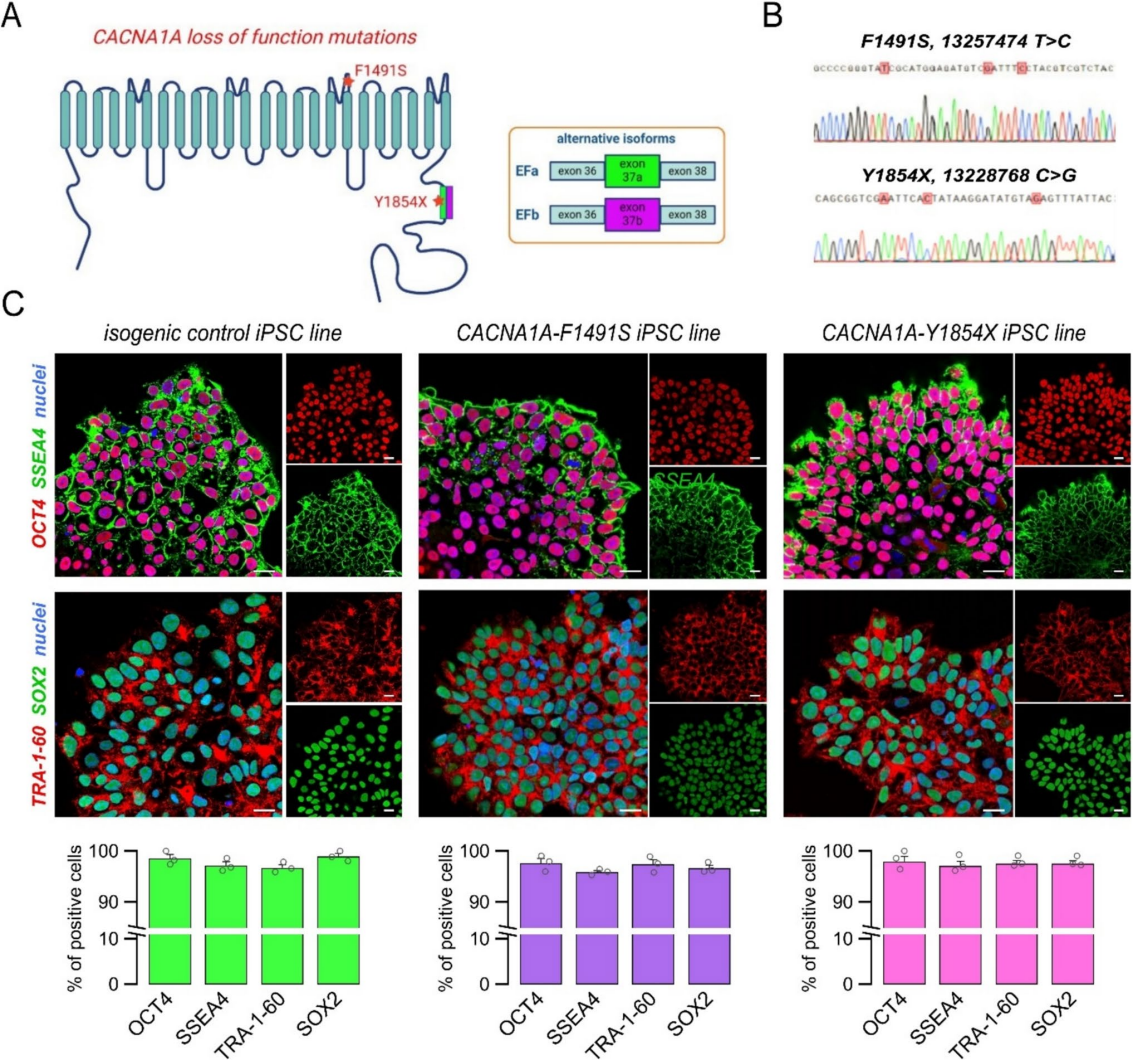
Isogenic iPSC lines carrying *CACNA1A* loss-of-function mutations. (**A**) Cartoon showing Ca_V_2.1 channel topology with the position of F1491S and Y1854X mutations. F1491S is located in a domain coded by a constitutive exon and therefore affects all *CACNA1A* isoforms. Y1854X is located in a C-terminal domain coded by the alternative exon 37a and selectively affects the Ca_V_2.1[EFa] isoform. (**B**) Sanger sequencing results from iPSC lines in which the indicated mutations were introduced by CRISPR/Cas9 genome editing. Silent mutations in the gRNA binding site were also introduced to prevent re-cutting. (**C**) Representative confocal images (top) and summary graphs (bottom) showing the analysis of undifferentiated state markers in isogenic control and mutated iPSC lines. Scale bar: 10 μm. The typical stem cell markers OCT4, SSEA4, SOX2, and TRA-1–60 were quantified by immunofluorescence. At least 2000 cells were analyzed for each marker and sample at the cell passages preceding the neural induction. The bar graphs show data as mean ± SEM, while single biological replicates are superimposed as dots (*n* = 3)

To model *CACNA1A* deficiency, we selected two *CACNA1A* loss-of-function mutations that induce severe forms of EA2 (Fig. 1A) [37, 41]. The F1491S mutation is located in a domain coded by a constitutive exon and therefore affects all *CACNA1A* isoforms. In contrast, the Y1854X mutation is located in the proximal C-terminal domain coded by the mutually exclusive exon 37a and therefore affects only the isoforms containing this exon (Ca_V_2.1[EFa]) and not those containing the mutually exclusive exon 37b (Ca_V_2.1[EFb]; Fig. 1A). Both mutations were been previously reported to abolish Ca_V_2.1 calcium channel activity [37, 41]. However, while the loss-of-function effect of the nonsense mutation can be directly attributed to the premature termination codon in a functional domain of the protein, the mechanism behind the loss-of-function caused by F1491S remains unclear. To investigate this, we performed heterologous expression experiments in HEK-293 cells, followed by cell surface biotinylation and Western blot analysis. We found comparable expression levels for wild type and F1491S Ca_V_2.1 in total cell lysates. However, the expression of the mutant protein was significantly reduced in the plasma membrane fraction (Fig. S1), suggesting that the F1491S mutation impairs Ca_V_2.1 trafficking to the cell surface.

To investigate the effect of the *CACNA1A* loss-of-function mutations on human neurogenesis, we generated a set of isogenic iPSC lines carrying either F1491S (Chr19:13,257,474 T > C) or Y1854X (Chr19:13,228,768 C > G) by means of CRISPR/Cas9 genome editing technology (Fig. 1B-C). All the iPSC lines were validated by Sanger sequencing to check for the presence of the desired mutations, array-CGH to verify the genomic stability after the genome editing and clonal selection processes, and by immunofluorescence detection and analysis of classical stem cell markers (OCT4, SSEA4, TRA-1–60, SOX2). Each edit resulted in homozygous mutations, and all iPSC lines retained genomic integrity and displayed homogenous expression of the markers of undifferentiated state (Fig. 1B,C).

### *CACNA1A* mutations affect *CACNA1A* expression in iPSC-derived neural cells

**Fig. 2:**
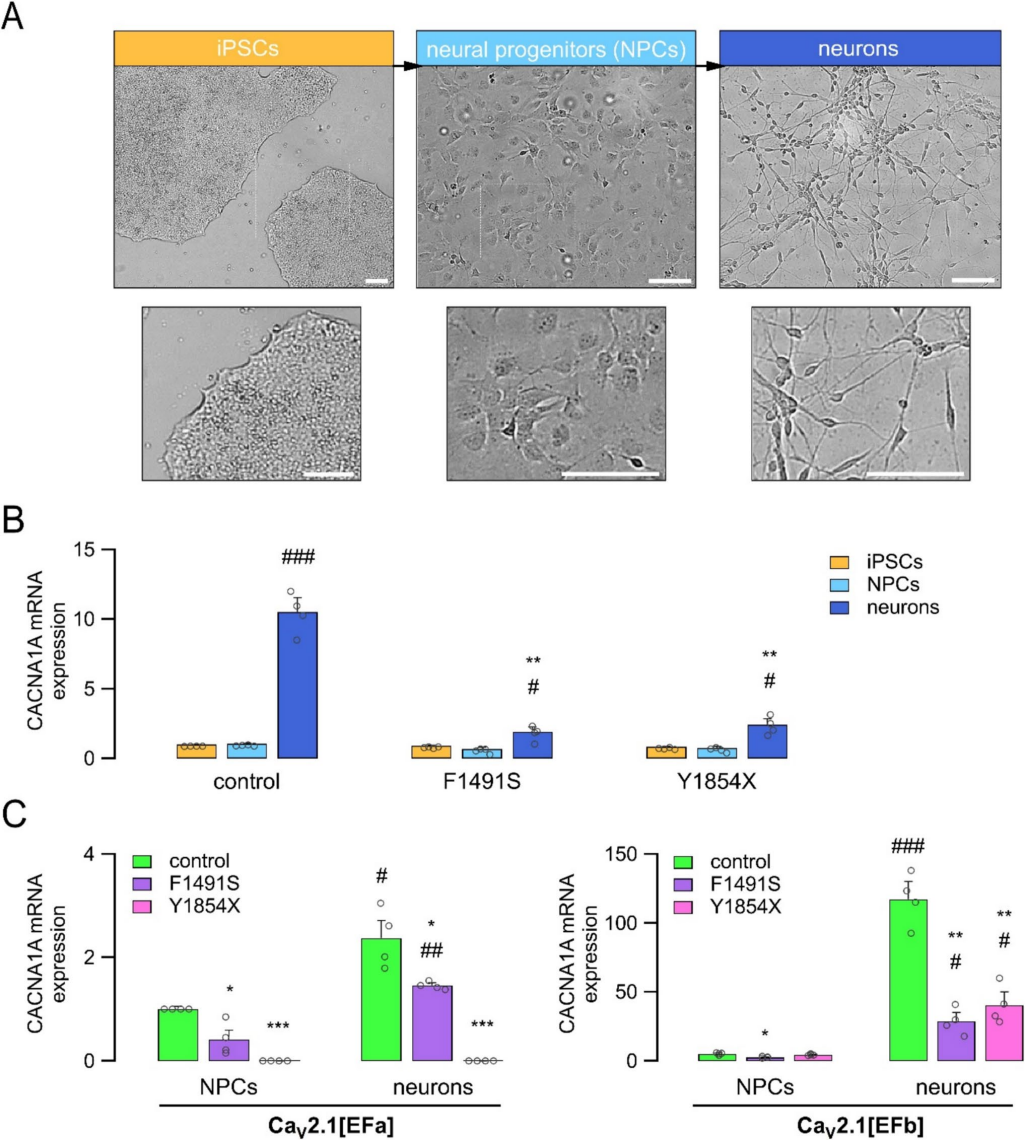
*CACNA1A* mutations affect *CACNA1A* transcripts expression in iPSC-derived NPCs and neurons. (**A**) Representative brightfield images (top) and enlarged regions (bottom) showing typical morphology of control iPSC colonies, neural progenitors (NPCs), and neurons. Scale bar: 100 μm. (**B**) Quantification of *CACNA1A* mRNA by real time qPCR in control and mutated iPSCs, NPCs, and neurons (42 DIV). Data were normalized by the expression of PPIA and RPL13 A as reference genes, and with isogenic control iPSCs as reference sample. Data are presented as mean ± SEM (bars) and single replicates (dots). **, *p* < 0.01 vs control (Student’s t test, *n* = 4); #, *p* < 0.05; ###, *p* < 0.001 neurons vs NPCs (Student’s t test, *n* = 4). (**C**) Quantification of Ca_V_2.1[EFa] (left) and Ca_V_2.1[EFb] (right) alternative isoforms by real time qPCR using isoform-specific primers in control and mutated NPCs and neurons (42 DIV). Data were normalized by the expression of PPIA and RPL13 A as reference genes, and with control NPCs as reference sample. Data are presented as mean ± SEM (bars) and single replicates (dots). *, *p* < 0.05; **, *p* < 0.01; ***, *p* < 0.001 vs control (Student’s t test, *n* = 4). #, *p* < 0.05; ##, *p* < 0.01; ###, *p* < 0.001 vs NPCs (Student’s t test, *n* = 4)

Isogenic control and mutated iPSC lines were used to generate neural models by differentiation protocols involving the sequential generation of embryoid bodies, neural rosettes, neural progenitors (NPCs), and neurons (Fig. 2A). Based on their proliferative and self-renewal capacity, the NPCs were expanded, cryopreserved, and tested between passages 1 to 4, prior to the final differentiation into neurons (Fig. 2A).

We first examined the expression of *CACNA1A* during the differentiation from iPSCs to neurons. In control cells, *CACNA1A* mRNA was detected at similar levels in iPSCs and NPCs and showed a 10-fold increase in neurons (Fig. 2B). Compared to isogenic control, no differences in *CACNA1A* expression were found in mutated iPSCs and NPCs, although a slight but not statistically significant decrease was observed in iPSCs carrying Y1854X and NPCs carrying either mutations (Fig. 2B). In contrast, *CACNA1A* expression was significantly reduced in F1491S and Y1854X neurons, which showed a 5- and 4-fold reduction, respectively, compared to control (Fig. 2B). Next, we investigated the expression of the alternative transcripts involving exons 37a and 37b. In control cells, both Ca_V_2.1[EFa] and Ca_V_2.1[EFb] variants were found already expressed in NPCs and increased in neurons, with Ca_V_2.1[EFb] being the predominant variant in both neurodevelopmental stages (Fig. 2C). Cells with F1491S showed reduced expression levels of both isoforms (Ca_V_2.1[EFa] mRNA levels in control and F1491S neurons were 2.38 ± 0.34 and 1.46 ± 0.05, respectively; *p* < 0.05, Student’s t test; Ca_V_2.1[EFb] mRNA levels in control and F1491S neurons were 117.29 ± 12.89 and 28.9 ± 6.2, respectively; *p* < 0.01; Student’s t test; Fig. 2C). In contrast, cells with the Y1854X mutation showed a nearly complete absence of the Ca_V_2.1[EFa] isoform suggesting that the premature stop codon in exon 37a induced non-sense mediated decay in these cells (Fig. 2C). For this mutant, the Ca_V_2.1[EFb] isoform was expressed at a similar level relative to control cells at the NPCs stage and reduced to about 35% in neurons (Ca_V_2.1[EFb] mRNA level in control and Y1854X neurons was 117.29 ± 12.89 and 40.47 ± 9.64, respectively; *p* < 0.05 with the Student’s t test; Fig. 2C).

### iPSC-derived NPCs carrying *CACNA1A* mutations show altered migratory capacity

**Fig. 3:**
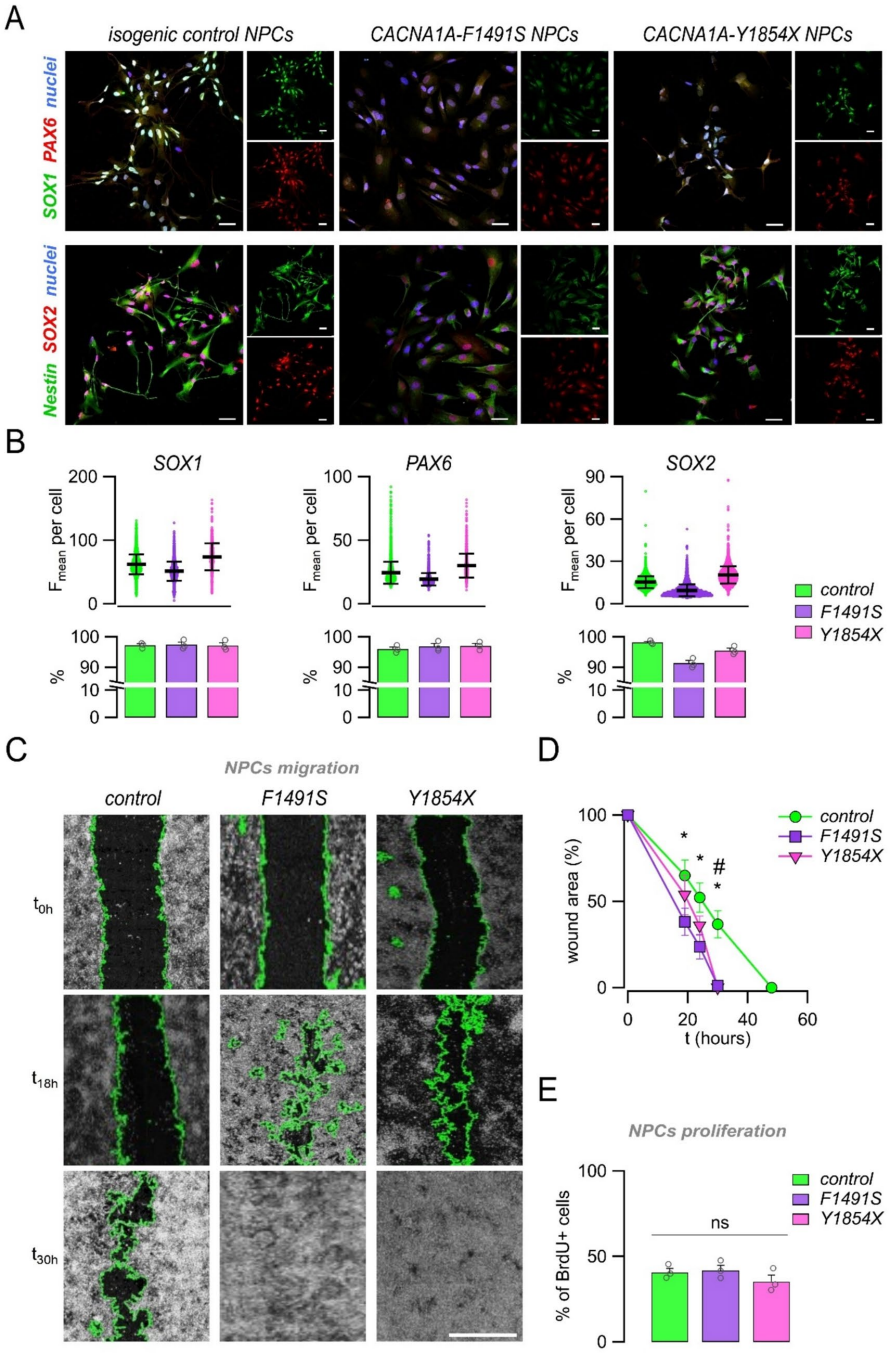
*CACNA1A* mutations alter the migratory capacity of NPCs. (**A**) Representative confocal images of control and mutated iPSC-derived NPCs showing the expression of typical neural progenitors’ markers: SOX1, PAX6, Nestin, and SOX2. Cells were also counterstained with DAPI to label cell nuclei. Scale bar: 20 μm. (B, top) Dot plot graphs showing the relative expression of the indicated nuclear markers quantified from immunofluorescence images at single-cell level (at least 2000 cells were analyzed for each marker and sample during 3 cell passages, from P2 to P4). (**B**, bottom) Bar graphs showing the percentage of positive NPCs for each marker and sample. Superimposed dots indicate biological replicates (*n* = 3). (**C**-**D**) Analysis of cell migration by wound healing assay. Representative images (**C**) and analysis (**D**) of wounded areas of confluent neural progenitors at the indicated post-wounding time points. Wound edges, detected by image segmentation analysis, are outlined in green. Scale bar: 500 μm. *, *p* < 0.05, F1491S vs control; #, *p* < 0.05, Y1854X vs control (Student’s t test, *n* = 3). (E) Graph showing the analysis of cell proliferation by the BrdU assay. Data are presented as mean ± SEM (bars) and single replicates (dots). No statistical significance was found with the Student’s t test by comparing each mutation with respect to control (*n* = 3)

Next, we assessed the expression of neural markers and the migratory capacity of NPCs (Fig. 3). By immunofluorescence combined with confocal microscopy and single-cell analysis of marker intensity, we found proper localization and high expression of SOX1, PAX6, SOX2, and Nestin in all NPC lines (Fig. 3A, B). To test the migratory capacity of NPCs, we performed a wound healing scratch assay on confluent NPCs cultures. Notably, F1491S-NPCs migrated significantly faster compared to control NPCs, as demonstrated by the nearly closed wound area just 18–24 h post-scratching (Fig. 3C, D). Y1854X-NPCs migrated slightly faster than control cells, with a significant different wound area at 30 h after scratch (Fig. 3C, D). To detect a possible contribution of different proliferative capacity, we also performed a proliferation assay. As reported in Fig. 3E, we found no detectable differences in the fraction of cells incorporating bromodeoxyuridine (BrdU) at 24 h after scratch. Taken together, these findings suggest that *CACNA1A* loss-of-function increases the migratory capacity of NPCs, particularly in the case of the F1491S variant.

### The F1491S, but not the Y1854X, mutation impairs neuronal induction and polarization

**Fig. 4:**
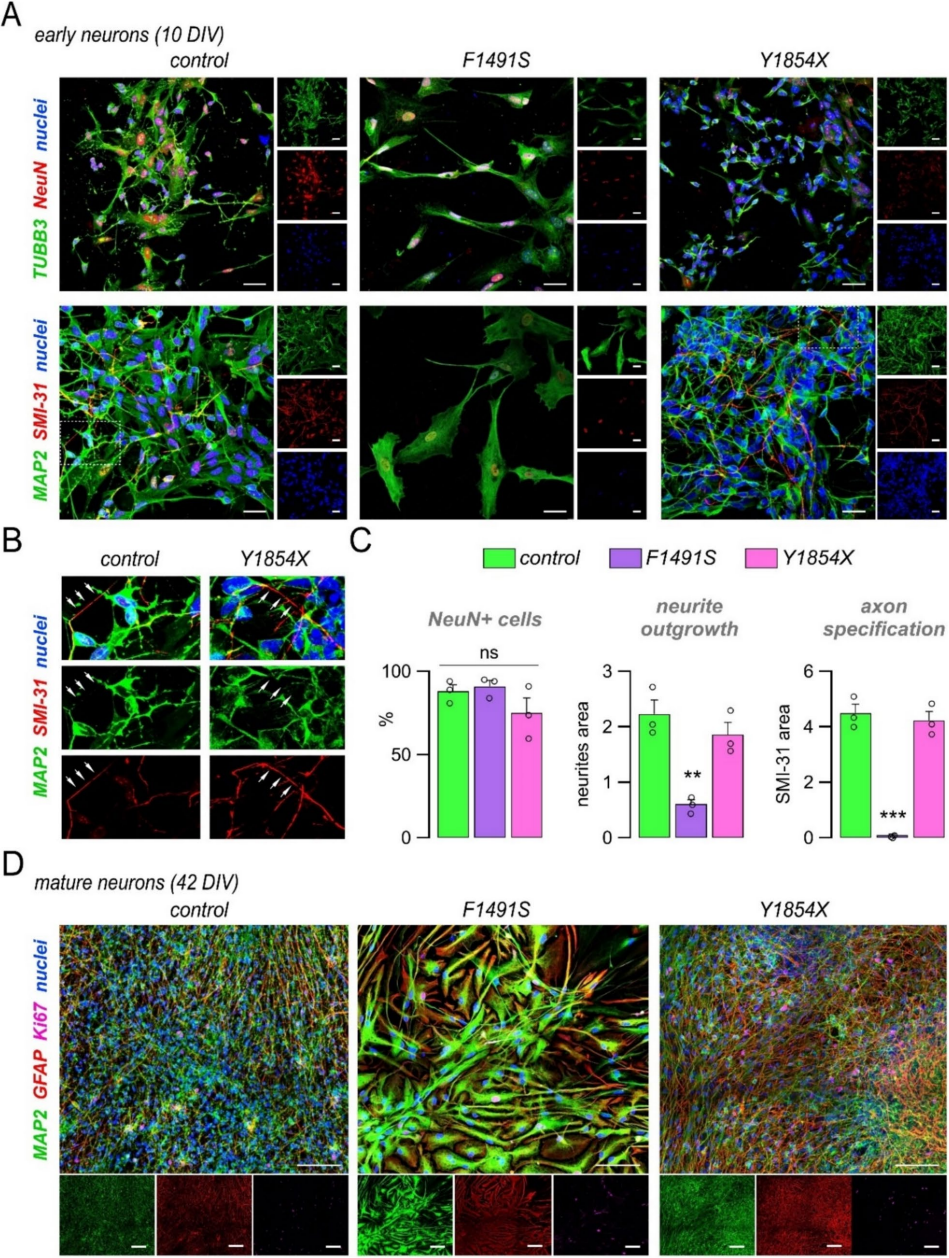
*CACNA1A* loss-of-function caused by F1491S, but not by Y1854X, impairs neuronal polarization and maturation. (**A**) Representative confocal images of control and mutated iPSC-derived neurons at 10 DIV. Cells were labeled with antibodies directed against TUBB3 and NeuN (top images) or MAP2 and SMI-31 (bottom images) as markers of neuronal maturation. Cells were also counterstained with DAPI to label cell nuclei. Scale bar: 20 μm. (**B**) Zoomed detail from images in panel A (dotted rectangles) showing MAP2 and SMI-31 staining in control and Y1854X neurons. White arrows indicate examples of MAP2-negative and SMI-31-positive axons. (**C**) Graphs showing the quantification of NeuN-positive cells (%), neurites outgrowth (neurites area), and axon specification (SMI-31 area) at 10 DIV. Data are shown as mean ± SEM (bars) and single replicates (dots). **, *p* < 0.01; ***, *p* < 0.001 vs control with the Student’s t test (*n* = 3). (**D**) Representative confocal images of control and mutated iPSC-derived neurons at 42 DIV. Cells were labeled with antibodies directed against MAP2, GFAP, and Ki67, as markers of neurons, glia, and proliferating cells, respectively. Cells were also counterstained with DAPI to label cell nuclei. Scale bar: 50 μm

To investigate neuronal induction and maturation processes, control and mutated NPCs were induced to differentiate in mixed neuronal cultures that were monitored at different developmental time points by immunofluorescence of various neuronal markers. As shown in Fig. 4, at an early differentiation stage (10 DIV, *i.e.*, 10 days of differentiation in vitro) all lines showed cytoskeletal and nuclear labeling for TUBB3 and NeuN (*RBFOX3*) with a comparable fraction of positive cells (Fig. 4A, B). At this stage, both control and Y1854X cells have started to polarize, developing typical neuronal-like morphology with distinct dendritic and axonal extensions, as evidenced by MAP2 and SMI-31 staining (Fig. 4A, B). At a more advanced stage of maturation (42 DIV), most control and Y1854X cells stained positive for markers of mature neurons (MAP2) or astrocytes (GFAP), thus forming an intricated network of neurons and glia, with only a minority of cells (< 1%) retaining positivity for the proliferation marker Ki67 (Fig. 4C). By contrast, cultures carrying the F1491S mutation presented less and abnormal cells. At 10 DIV, these cells appeared reduced in number and showed altered shapes with a larger soma and fewer cellular extensions (Fig. 4A). Apoptosis evaluation by Annexin-V and PI staining or by detection of cleaved caspase-3 revealed a higher number of apoptotic cells compared to control and Y1854X cultures (the percentage of apoptotic cells was 3.70 ± 0.49, 8.91 ± 1.77, and 3.60 ± 0.38 in control, F1491S, and Y1854X neurons, respectively, based on cleaved-caspase-3 detection; Fig. S2). Analysis of MAP2 and SMI-31 expression and localization revealed a significant reduction in neurites area (assessed by MAP2) and a lack of axonal labeling with the SMI-31 antibody, which instead stained the nucleus (Fig. 4A, B). At 42 DIV, cultures bearing the F1491S mutation still appeared largely divergent from control and Y1854X cultures, as indicated by their non-neuronal morphology and by the co-expression within the same cells of MAP2 and GFAP (Fig. 4C). These results indicate that the F1491S, but not the Y1854X, mutation induces cell loss and severe defects in neuronal induction, polarization, and neuron-to-glia specification.

To validate these findings, we searched for additional iPSC clones carrying the F1491S mutation to be differentiated into NPCs and neurons. Importantly, the altered migratory capacity of NPCs and the impaired generation of neurons was confirmed in a second F1491S clone (Fig. S3 and S4).

### The F1491S mutation alters the development of neural precursors

**Fig. 5:**
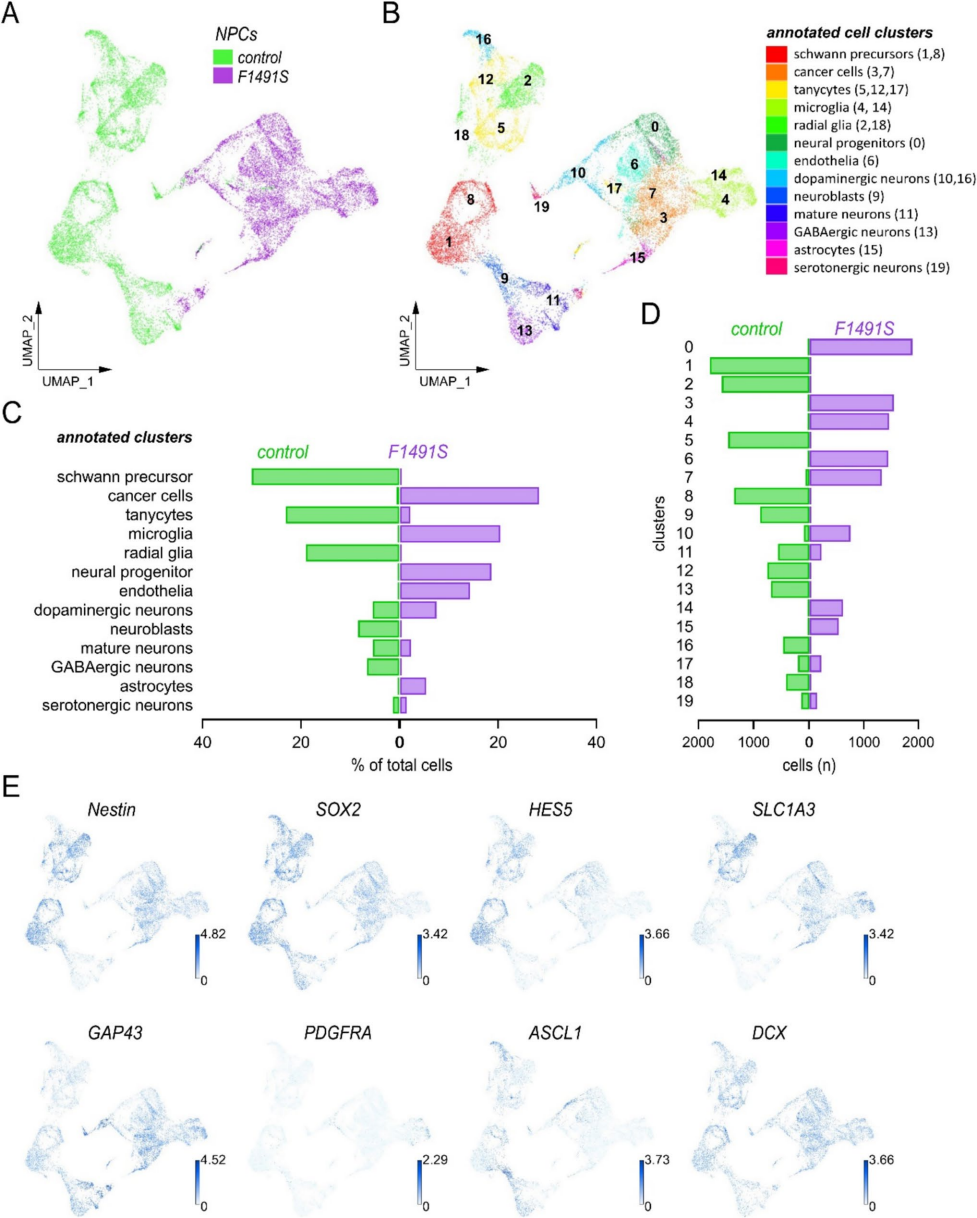
The F1491S mutation alters the development of neural precursors. Analysis of control and F1491S NPCs by scRNA-Seq revealed a divergent cell composition. (**A**, **B**) Global representation of gene expression through UMAP plot. About 10,000 cells were profiled for each group (10,490 and 10,166 control and F1491S NPCs, respectively). Each dot represents a single cell, whose position in the map reports the transcriptional similarity with respect to the neighbor cell. The different colors indicate the samples in (**A**), and the annotated ScType clusters in (**B**). (**C**) Graph showing the percentage of cells assigned to each annotated cell type. (**D**) Graph showing the number of cells assigned to each cluster. (**E**) Feature plots showing distribution and expression of the indicated genes as representative markers of neuroepithelial cells (*Nestin* and *SOX2*), radial glia (*HES5* and *SLC1A3*), Schwann precursor cells (*GAP43*), oligodendrocyte precursor cells (*PDGFRA*), intermediate progenitors (*ASCL1*), and immature neurons (*DCX*). Each dot represents a single cell and is colored according to expression level

To gain insight into the mechanisms underlying the early neurodevelopmental defects observed in cells carrying the F1491S mutation, we investigated the molecular signatures of NPC cultures by scRNA-seq. Strikingly, clustering and annotation of single-cell transcriptomes revealed a highly divergent cell composition of F1491S-NPCs compared to isogenic control-NPCs, particularly regarding the main types of neural stem and progenitor cells (Fig. 5A-D). Major markers of the annotated clusters are reported in Fig. S5 A, while the expression of selected neural markers is shown in Fig. 5E. Control NPCs resulted enriched for major cell types annotated as Schwann precursors, radial glia, and neuroblasts, which were completely absent in F1491S-NPCs (Fig. 5C-D). F1491S-NPCs were enriched for cell types annotated as cancer cells, microglia, neural progenitors, endothelia, and astrocytes, which were absent in control NPCs (Fig. 5C-D). Intrigued by the unexpected finding of cancer cells as one of the major annotated cell type in mutant cultures, we examined their specific markers identified by differential expression of scRNA-seq data. Among the markers with larger fold changes and larger differences in expression between pct.1 (percentage of cells where the gene is detected in the specific cluster) and pct.2 (percentage of cells where the gene is detected on average in the other clusters), we found many genes related to tumorigenesis, as for example *LHCGR*, *RGCC*, *CRHBP*, *FOSL1*, *HHEX*, and *ANKRD1*, but also genes normally expressed in neuronal or glial progenitors, such as *CAV1*, *NPY*, *MMP10*, and *SRFP4*, thus suggesting a mixed nature of cells in these clusters (Fig. S5B, C) [63–71]. We also looked at the hierarchical annotation procedure in ScType, which highlighted the various possible cell types considered for the annotation of clusters 3 and 7, including cancer cells, non-myelinating Schwann cells, cancer stem cells, endothelial cells, glutamatergic neurons, astrocytes, and Schwann precursors (Fig. S5 C). The cancer cells type was the one with the highest score and therefore was selected for cell type assignment (Fig. S5 C). Control and F1491S-NPCs also shared some cell types, annotated as tanycytes, mature neurons, dopaminergic, and serotonergic neurons, although with different relative abundance and a general low transcriptional similarity (*i.e.,* such clusters had the same annotations but were distant from each other in the two-dimensional UMAP map) (Fig. 5A-D). To better investigate their transcriptional state, we combined scRNA-seq data with computational analyses and depicted the genetic regulatory network (GRN) of control and F1491S NPCs. GRN analysis highlighted regulons involved in cell cycle regulation (E2F8) and cell proliferation (ERF), which are activated in different clusters of both control and mutated NPCs, with higher activity in the latter (Fig. S6 A-C). Other regulons were found specifically activated in control or mutated cells (Fig. S6B). The regulon with the highest activity score in control NPCs was JUND, whereas CREB3L1 was the most activated in mutated cells, particularly in those clusters annotated as microglia (Fig. S6B, C).

Taken together, these findings indicate that F1491S-induced *CACNA1A* loss-of-function severely disrupts human neurogenesis during early neural induction stages by altering the development, specification, and state of neural progenitor cells.

### The Y1854X loss-of-function mutation impairs neuronal network synchronization and composition

**Fig. 6:**
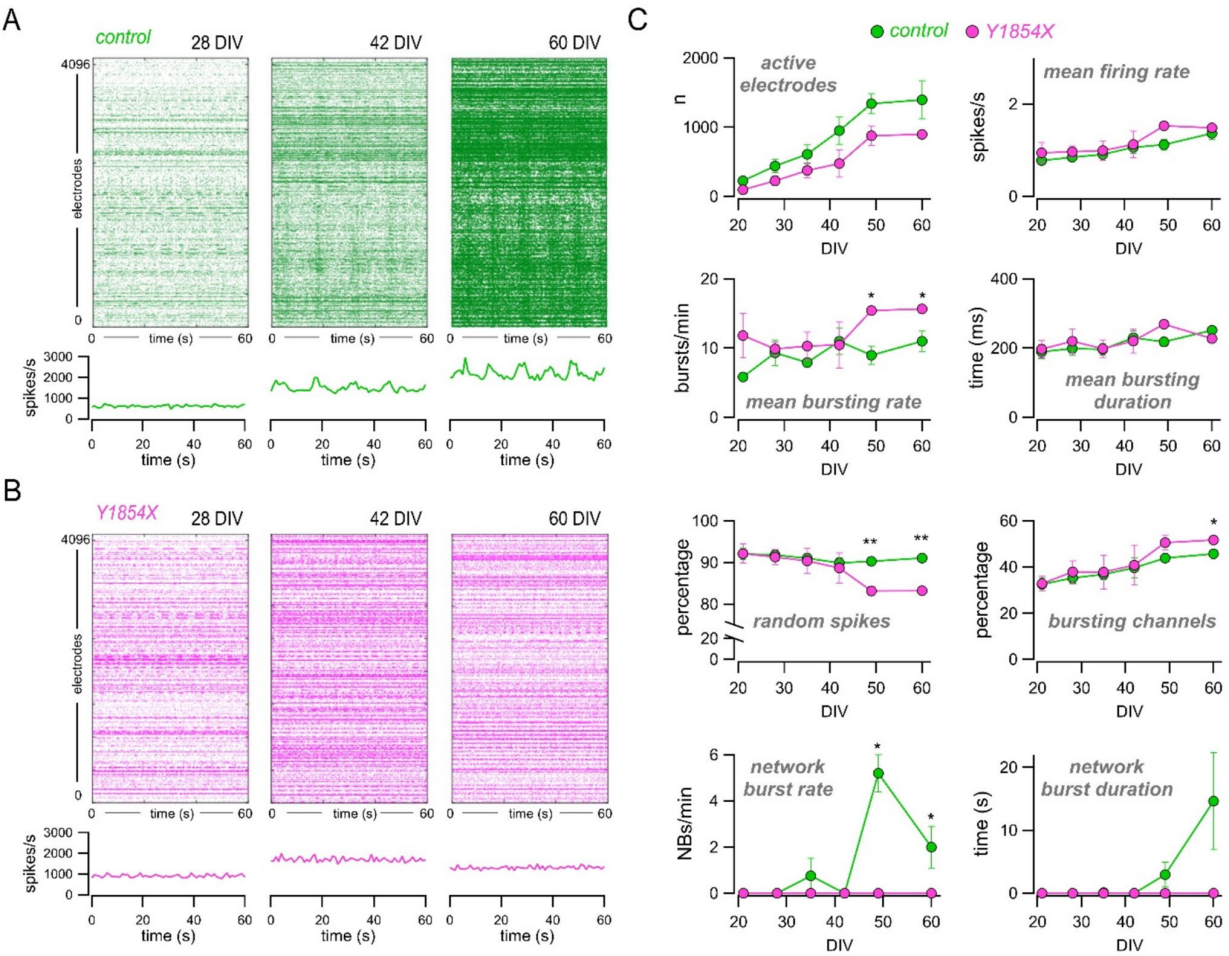
*CACNA1A* loss-of-function caused by Y1854X mutation impairs neuronal network synchronization. (**A**, **B**) Raster plots (top) and array wide firing rate graph (bottom) showing 60 s of spontaneous activity recording at 28, 42 and 60 DIV in control (**A**) and Y1854X (**B**) neuronal cultures. The raster plot y-axis represents electrodes (from 0 to 4096), and each dot indicates a detected spike. The array wide firing rate quantifies the level of activity shown in the above raster plots and highlights the appearance of synchronous network events only in the control (green peaks at 42 and 60 DIV). (**C**) Graphs comparing the activity of control and mutated iPSC-derived neurons in terms of number of active electrodes, mean firing rate, mean bursting rate, mean burst duration, percentage of random spikes, percentage of bursting channels, network burst rate, and network burst duration. Data are shown as mean ± SEM. *, *p* < 0.05; **, *p* < 0.01 vs control (Student’s t test, *n* = 3–6 replicates for each time points and sample)

**Fig. 7:**
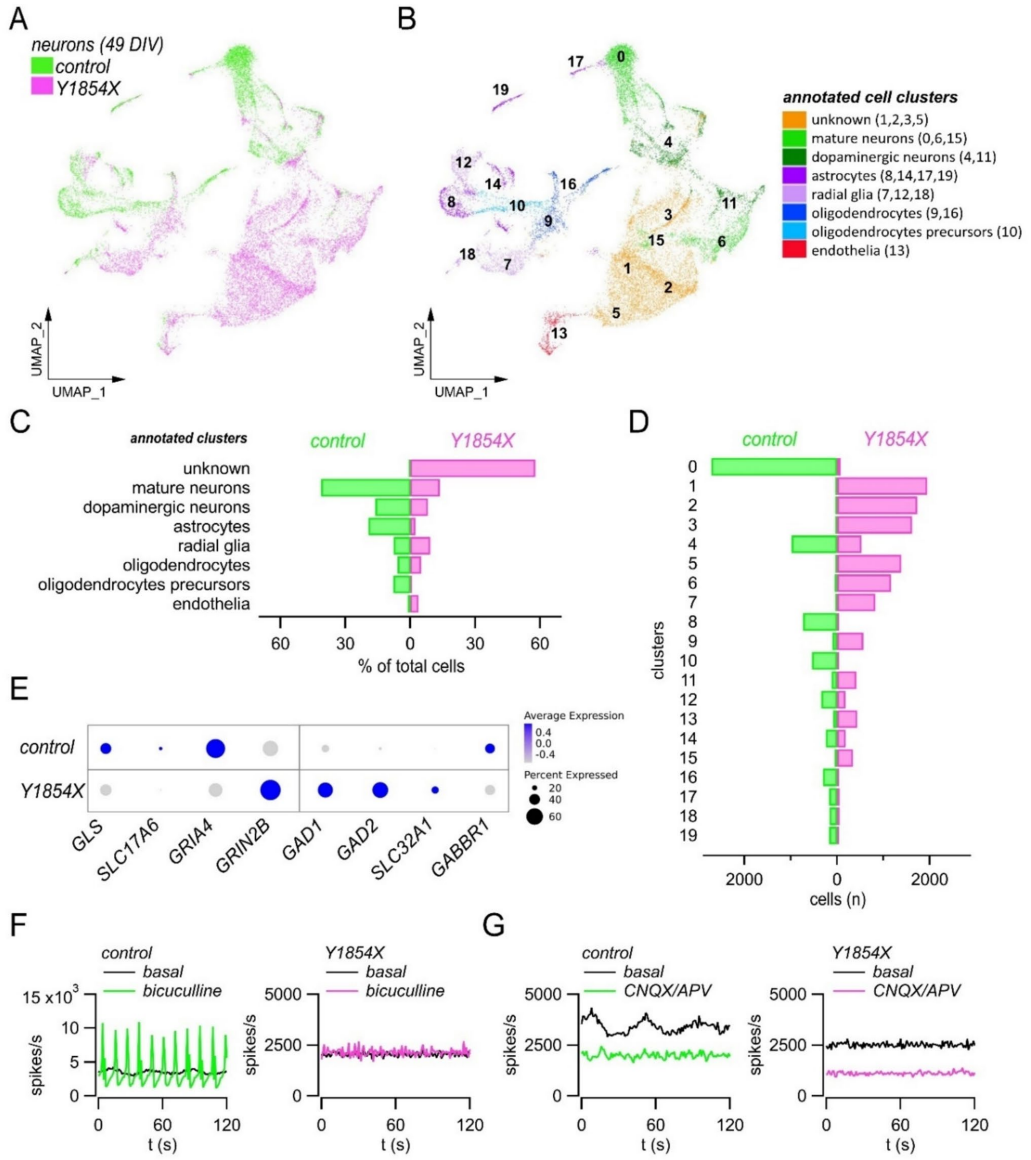
*CACNA1A* loss-of-function caused by Y1854X mutation alters neuronal network composition. (**A**, **B**) Global representation of gene expression through UMAP plot. 6815 and 11,551 cells were profiled for control and Y1854X neuronal cultures, respectively. Each dot represents a single cell, whose position in the map indicates the transcriptional similarity with respect to the neighbor cell. The different colors indicate the samples in (**A**), and the annotated ScType clusters in (**B**). (**C**) Graph showing the percentage of cells assigned to each annotated cell type. (**D**) Graph showing the number of cells assigned to each cluster. (**E**) Dot plot showing the expression of the indicated genes in control and Y1854X neurons. Dot color intensity represents the average expression of the indicated genes, and dot size represents the percentage of cells expressing those genes. (**F**) AWFR graphs showing 2 min-recording of spontaneous activity under basal condition and after administration of 30 μM bicuculline in control and Y1854X cultures. (**G**) AWFR graphs showing 2 min-recording of spontaneous activity under basal condition and after administration of 50 μM CNQX and 80 μM APV in control and Y1854X cultures

In contrast to F1491S, the Y1854X loss-of-function mutation apparently leads to normal neuronal induction and maturation, at least at a cell morphology level, as revealed by the proper polarization and differentiation of cells carrying this premature stop-codon variant selectively affecting the Ca_V_2.1[EFa] isoform. We further investigated Y1854X-neurons at the functional level by electrophysiological analysis. Control and mutated neurons were cultured on high-density MEA (HD-MEA), and their spontaneous electrical activity was recorded once every week between 21 and 60 DIV. As shown in Fig. 6A-C, both control and mutated neuronal cultures developed spontaneous activity, as revealed by the increasing spike detection rate (Fig. 6A-C). The number of active electrodes rose from 225 ± 51.19 (mean ± SEM) and 97 ± 24 at 21 DIV to 1395.5 ± 275.83 and 898.25 ± 25.6 at 60 DIV in control and mutated neurons, respectively; the mean firing rate increased from 0.78 ± 0.04 and 0.94 ± 0.22 at 21 DIV to 1.37 ± 0.13 and 1.49 ± 0.03 at 60 DIV in control and mutated neurons, respectively (Fig. 6C). No statistically significant differences were found in these parameters between control and mutated neurons. The mean bursting rate was initially comparable between control and mutated neurons, and then diverged with mutated cultures showing a significantly higher rate at 49 and 60 DIV (the mean bursting rate at 49 DIV was 8.96 ± 1.32 and 15.41 ± 0.12 in control and mutated neurons, respectively, and at 60 DIV was 10.99 ± 1.47 and 15.66 ± 0.39 in control and mutated neurons, respectively; *p* < 0.05 with the Student’s t test) (Fig. 6C). The mean bursting duration was similar (≈ 200 ms) in control and mutated neurons at all time points (Fig. 6C). There were differences with respect to the percentage of random spikes and bursting channels (electrodes), which were lower and higher, respectively, in mutated cultures (Fig. 6C). Most importantly, network-wide synchronized bursting activity appeared in control but not in mutated samples, revealing a complete lack of network synchronization in Y1854X cultures (Fig. 6A-C). Coordinated network activity relies on many factors, including proper neuronal maturation, network composition, and balance of excitatory and inhibitory inputs. To investigate which of these factors could have prevented the onset of synchronized activity in mutated samples, we applied scRNA-seq analysis to control and Y1854X neuronal cultures at 49 DIV. As shown in Fig. 7, in control cultures, mature neurons and astrocytes were identified as the major cell types, contributing to about 60% and 20% of total cells (3909 and 1309 cells annotated as neurons and astrocytes, respectively, out of 6815 total cells). Minor cell clusters were annotated as radial glia (7.7% of total cells), oligodendrocytes precursors (7.9%), oligodendrocytes (5.9%), and endothelial cells (1.2%) (Fig. 7A-D). In contrast, in Y1854X cultures, the majority of cells (57.8%) failed to be annotated as a specific brain cell type (“unknown” clusters in Fig. 7A-C), whereas mature neurons and astrocytes were less represented, accounting for only 21.7% and 2.4% of total cells, respectively (Fig. 7A-D). Cells annotated as radial glia (9.2%) and oligodendrocytes (5.0%) were identified in similar proportions compared to controls, whereas oligodendrocytes precursors were reduced (0.2%) and endothelial cells were increased (3.7%) (Fig. 7B-D). The GRN analysis confirmed the different cell composition and transcriptional state by highlighting various regulons that are differently activated in control and mutated neuronal cultures (Fig. S7 A). The regulon with the highest activity score in control neurons was BHLHE41, whereas GSX2, E2 F8, STAT1, ELF1, JUND, and JUNB were the most activated regulons in mutated cells (Fig. S7 A-C).

To gain insight into the molecular identity of the cell types annotated as “unknown” (including cell clusters 1, 2, 3, and 5), we examined their specific markers identified by differential expression of scRNA-seq data (Fig. S8). Interestingly, among the markers with larger fold changes and larger differences in expression between pct.1 and pct.2, we found many genes linked to the development, maintenance, and function of GABAergic neurons: *DLX1/2/5*, *KLHL35*, *NR2F2*, *CXCR4*, *CALB2*, *SCGN*, *SP8*, *PDZRN3*, *ST18*, *THRB*, *WNT5A*, *ADARB2*, *IGFBPL1*, *CCND2*, *TAC3*, *GABRG3*, and *GABRB2* (Fig. S8B) [72–90]. We also examined the expression of other classical markers of inhibitory neurons and found *GAD1*, *GAD2* and *SLC32A1* (*VGAT*) expression particularly concentrated in the UMAP plot regions corresponding to the unknown clusters 1, 2, and 5 (Fig. S9 A-C and Fig. 7E). The same regions were enriched for markers of immature neurons, as *DCX* and *TUBB3*, and displayed low expression of *SYP*, a marker of mature neurons (Fig. S9D). These expression profiles suggest that cells belonging to the unknown clusters represent immature, or not-fully specified, GABAergic interneurons. Moreover, we examined the expression of classical glutamatergic markers, such as *GLS* and *SLC17A6* (*VGLUT2*), and found that they were much less represented in mutated cultures both in terms of average expression and percentage of expressing cells (Fig. 7E and Fig. S9E). Together with the reduced number of mature neurons and astrocytes, these findings highlight an altered neural network composition and maturation caused by the Y1854X-CACNA1A loss-of-function mutation.

The higher proportion of GABAergic interneurons indicated by single-cell transcriptomic data may contribute to the lack of network burst activity in mutant cultures by unbalancing the excitation-inhibition ratio (Fig. 7E and Fig. S9 C, E). To test this hypothesis, we recorded the spontaneous electrical activity after administration of bicuculline or CNQX/APV to block GABA- or AMPA/NMDA- receptors, respectively. In control networks, bicuculline significantly boosted, while CNQX/APV abolished, the synchronized electrical activity (Fig. 7F, G). In mutated networks, CNQX/APV reduced the spike detection rate similarly to controls, whereas bicuculline treatment was ineffective (Fig. 7F, G). This lack of response to disinhibition suggests a reduced maturation also of the excitatory component of mutated networks, as also indicated by scRNA-seq data.

## Discussion

In this study, we explored the molecular and functional properties of iPSC-derived neural models to investigate the role of *CACNA1A* in human neurogenesis and to uncover the cellular development mechanisms underpinning neurological disorders caused by *CACNA1A* deficiency. Our experimental design involved the generation of isogenic human iPSCs by CRISPR/Cas9 genome editing, enabling the investigation of *CACNA1A* mutations in a shared genetic background [91]. We selected two *CACNA1A* loss-of-function mutations that induce severe forms of EA2 and have been previously described to abolish Ca_V_2.1 calcium channel activity [37, 41]. To model human neurodevelopmental processes in vitro and investigate the effects of *CACNA1A* mutations, we adopted neural differentiation protocols based on the sequential generation of intermediate precursors and neuronal networks. As revealed by single-cell transcriptomic analysis and automated cell type annotation, we obtained heterogeneous neural cell populations. Indeed, control NPCs cultures were composed of different types of neural precursors, including radial glia, Schwann precursors, and neuroblasts, and some immature neurons committed to various lineages (glutamatergic, dopaminergic, GABAergic, and serotonergic). Upon terminal differentiation, NPCs generated a mixed network composed of diverse neuronal (glutamatergic, GABAergic, and dopaminergic) and glia (astrocytes and oligodendrocytes) subtypes. Importantly, control neuronal networks developed spontaneous and coordinated electrophysiological activity, indicating the achievement of functional maturation.

The heterogeneous composition of these cultures is in agreement with recent data obtained in ex-vivo tissues and in vitro models, including 2D cultures generated through similar protocols and cerebral organoids [92–95]. Since such heterogeneous nature reflects the wide variety of cell types present in the human neocortex, these models appear particularly useful to investigate the cell mechanisms of genetic neurodevelopmental disorders, also offering the possibility to take into consideration the complex and mutual cell–cell interactions which drive the proper development of brain cells. Indeed, such features cannot be accurately modeled in simpler neuronal cultures composed of a single cell type and may be disrupted in co-culture approaches combining differentiated cells derived from different species or with different genetic background.

By combining these human iPSC-derived neuronal models with single-cell transcriptomics and HD-MEA electrophysiology, we uncovered a previously unrecognized essential role for *CACNA1A* in early stages of neural induction, specification, and maturation. Additionally, we found that different *CACNA1A* loss-of-function mutations produced distinct neurodevelopmental deficits. *CACNA1A* loss-of-function had a profound impact on the transcriptional landscape of neural cells, as shown by scRNA-seq. The F1491S loss-of-function mutation, which affects all Ca_V_2.1 splice isoforms, hindered the proper development and specification of neural precursors, which were depleted in the radial glia fraction and enriched in cancer and microglial cells. The premature stop codon mutation Y1854X, which selectively affects the Ca_V_2.1 subpopulation containing the mutually exclusive exon 37a, altered the maturation and composition of neuronal networks, which showed a large number of cells resembling immature neurons, an increased induction of GABAergic interneurons over excitatory neurons, and a reduced number of astrocytes. These alterations in networks composition, together with the known role of Ca_V_2.1 in sustaining synaptic transmission [3, 4], may explain the lack of synchronized activity observed in mutated cultures.

Previous studies reported that Ca_V_2.1 loss-of-function in mice produces neurological dysfunctions, including ataxia, dystonia, and epilepsy, mostly by means of development-independent mechanisms [96–105]. Indeed, overlapping phenotypes have been observed in animal models with constitutive (inborn) and conditional (adult-onset) *Cacna1a* ablation [96–106]. Moreover, it has been shown that Ca_V_2.1 channels, together with Ca_V_2.2 and Ca_V_2.3, are essential for action potential triggered release but are dispensable for synapses assembly in cultured mouse neurons [107]. Thus, Ca_V_2.1-related neurological dysfunctions have been generally associated with altered synaptic transmission within various brain circuits [3, 96–105]. Notably, our findings indicate that Ca_V_2.1 may exert relevant roles beyond the regulation of neurotransmitters release and highlight a wider involvement of *CACNA1A* in early neurodevelopmental mechanisms, at least in human cells.

A number of direct and indirect ways through which *CACNA1A* loss-of-function may affect the developmental fate and the maturation of neural cells can be proposed and may involve, for example, the well-known role of calcium signaling in controlling gene expression. For example, it has been demonstrated that loss of calcium flux through other voltage-gated calcium channels, as Ca_V_1.2 and Ca_V_2.2, affects not only the synaptic transmission but also alters various calcium signaling pathways and downstream processes shaping nuclear gene expression and differentiation [108–111]. In this regard, the previously reported expression of *CACNA1A* in different types of neurons (including glutamatergic neurons and parvalbumin-expressing GABAergic interneurons) as well as in astrocytes [112–115] suggests that its loss-of-function may influence the proper function and maturation of these different cellular components involved in neuronal network formation. This aligns with the altered development of excitatory and inhibitory neurons, as well as astrocytes found in our study. *CACNA1A* could also directly contribute to the transcriptional regulation of other neural genes by means of α1ACT, a secondary protein of the *CACNA1A* mRNA that functions as a transcription factor and was found to orchestrate the dynamic gene expression involved in early development of the cerebellum [48, 116]. Moreover, our gene regulatory network analysis revealed the activation of transcriptional pathways involved in ER-stress response, as those controlled by CREB3L1, in cells carrying the F1491S mutation. As this mutation impairs Ca_V_2.1 trafficking to the cell surface, it is tempting to speculate that accumulation of mutated Ca_V_2.1 in intracellular compartments may have induced cytotoxicity and activation of signaling pathways which contributed to the altered development of neural cells. Notably, transcriptomic alterations have also been described in Nagoya and tottering mice carrying Ca_V_2.1 mutations [108, 117, 118].

The broad spectrum of *CACNA1A*-related neurological disorders relies on the large number of different disease-causing variants. To date, more than 3700 *CACNA1A* variants are reported in ClinVar, with about 560 listed as Pathogenic or Likely Pathogenic. Such variants can be detected in monoallelic or biallelic conditions and, on a functional point of view, can exert different effects on Ca_V_2.1 channel encompassing both gain-of-function and loss-of-function through different mechanisms. The high molecular complexity of Ca_V_2.1 channels, which exist in different complexes and forms based on the assembling of different subunits and splicing isoforms, may also contribute to the variable effects of the mutations and thus to the variable genotype–phenotype relationships. For example, many studies have highlighted the diverse function of *CACNA1A* isoforms produced by the alternative splicing of the mutually exclusive exons 37a and 37b [4, 5, 7, 9, 13–16]. In this regard, we found cell phenotypes that correlated with dosage- and isoform-dependent Ca_V_2.1 deficiency. Since we introduced both *CACNA1A* mutations in homozygous conditions, the F1491S mutation, by affecting all isoforms, induced a complete loss-of-function of Ca_V_2.1 channels, whereas the Y1854X mutation, by leaving the Ca_V_2.1[EFb] isoform unaltered, produced a selective loss-of-function of the Ca_V_2.1[EFa] isoform. Cells carrying the F1491S mutation showed a more severe phenotype involving an early impairment of neurogenesis, as demonstrated by the altered development of neural precursors, which impeded their differentiation into neurons. In contrast, cells with the Y1854X mutation produced electrophysiological active neuronal cultures which, however, failed to mature into networks with coordinated activity. Such findings suggest that functional Ca_V_2.1[EFb] channels are somewhat sufficient to sustain early neuronal development and agree with the previously described predominant role of Ca_V_2.1[EFb] over Ca_V_2.1[EFa] during early brain development in rodents [13, 14]. However, Ca_V_2.1[EFa], which is the more effective isoform in sustaining synaptic transmission [4, 13, 14, 16], is still required for the complete maturation of neuronal networks and the onset of synchronized activity.

In conclusion, our findings reveal previously unrecognized roles of *CACNA1A* in neural induction, neuronal differentiation and maturation, and highlight the differential contribution of the divergent variants Ca_V_2.1[EFa] and Ca_V_2.1[EFb] in the development of human neuronal cells. Future studies will have to elucidate if these findings in vitro align with the systemic impact of *CACNA1A* loss-of-function, particularly taking into consideration the large number of disease-causing mutations and the diverse inheritance models and will have to explore novel therapeutic strategies based, for example, on the rescue of *CACNA1A* or the modulation of alternative targets to circumvent *CACNA1A* deficiency. In this regard, the iPSC-derived models developed in this study may provide the foundation for future testing of potential therapeutic approaches for *CACNA1A*-related neurological disorders.

## Supplementary Information

Below is the link to the electronic supplementary material.

Supplementary file1 (PDF 1.19 MB)

## Data Availability

Single-cell RNA-seq data have been deposited at GEO and are publicly available as of the date of publication. Accession numbers are listed in the resources table. Other data reported in this paper will be shared by the lead contact upon request.
