# Supplementary material for: CACNA1A loss-of-function affects neurogenesis in human iPSC-derived neural models
Source: Cell Mol Life Sci. 2025 Jun 14;82(1):234. doi: 10.1007/s00018-025-05740-7 (PMC12165946; doi:10.1007/s00018-025-05740-7)
Supplement: Supplementary file 1 — Supplementary file1 (PDF 1.19 MB) [file 18_2025_5740_MOESM1_ESM.pdf]

Supplementary information
for

CACNA1A loss-of-function affects neurogenesis in human iPSC-derived neural models

Ilaria Musante[1], Davide Cangelosi[2], Lorenzo Muzzi[3], Fanny Jaudon[4], Marco Di Duca[1], Sara Guerrisi[1], Francesca Antonini[5], Yeraldin Chiquinquira Castillo De Spelorzi[6], Lorenzo A. Cingolani[4], Federico Zara[1,3,*], Paolo Scudieri[1,3,*]

[1] Medical Genetics Unit, IRCCS Istituto Giannina Gaslini, Genoa, Italy
[2] Clinical Bioinformatics Unit, IRCCS Istituto Giannina Gaslini, Genoa, Italy
[3] Department of Neurosciences, Rehabilitation, Ophthalmology, Genetics, Maternal and Child Health (DiNOGMI), University of Genoa, Genoa, Italy
[4] Department of Life Sciences, University of Trieste, Trieste, Italy
[5] Core facilities for Omics Science, IRCCS Istituto Giannina Gaslini, Genoa, Italy.
[6] Genomics Facility, Italian Institute of Technology (IIT), Genoa, Italy

[*] Correspondence: paolo.scudieri@unige.it, federico.zara@unige.it

Resources table

REAGENT or RESOURCE	SOURCE	IDENTIFIER
**Antibodies**		
OCT4 Recombinant Rabbit Monoclonal Antibody (3H8L1.12)	Thermo Fisher Scientific	Cat# 703927, RRID:AB_2827381
SSEA4 Monoclonal Antibody (MC-813-70)	Thermo Fisher Scientific	Cat# MA1-021, RRID:AB_2536687
TRA-1-60 Monoclonal Antibody (TRA-1-60)	Thermo Fisher Scientific	Cat# MA1-023, RRID:AB_2536699
SOX2 Monoclonal Antibody (Btjce), eBioscience	Thermo Fisher Scientific	Cat# 14-9811-82, RRID:AB_11219471
SOX2 Polyclonal Antibody	Thermo Fisher Scientific	Cat# PA1-094X, RRID:AB_2539863
SOX1 Recombinant Rabbit Monoclonal Antibody (JJ20-40)	Thermo Fisher Scientific	Cat# MA5-32447, RRID:AB_2809724
PAX6 Monoclonal Antibody (13B10-1A10)	Thermo Fisher Scientific	Cat# MA1-109, RRID:AB_2536820
Nestin Monoclonal Antibody (2C1.3A11)	Thermo Fisher Scientific	Cat# MA1-5840, RRID:AB_1077111
Anti-beta3-tubulin	Synaptic Systems	Cat# 302 304, RRID:AB_10805138
Anti-NeuN	Sigma Aldrich	Cat# MAB377, RRID:AB_2298772
Anti-MAP 2	Synaptic Systems	Cat# 188 004, RRID:AB_2138181
Purified anti-Neurofilament Marker	BioLegend	Cat# 837904, RRID:AB_2566782
Anti-GFAP	Synaptic Systems	Cat# 173 011, RRID:AB_2232308
Anti-Ki67 antibody	Abcam	Cat# ab15580, RRID:AB_443209
BrdU antibody [BU1/75 (ICR1)]	Abcam	Cat# ab6326, RRID:AB_305426
Anti-β-Tubulin III	Sigma Aldrich	Cat# T2200 RRID: AB_262133
anti-Caspase-3	R&D System	Cat# AF835 RRID: AB_2243952
Goat anti-Mouse IgG1 Cross-Adsorbed Secondary Antibody, Alexa Fluor™ 555	Thermo Fisher Scientific	Cat# A-21127, RRID:AB_2535769
Goat anti-Rabbit IgG (H+L) Cross-Adsorbed Secondary Antibody, Alexa Fluor™ 488	Thermo Fisher Scientific	Cat# A-11008, RRID:AB_143165
Goat anti-Rabbit IgG (H+L) Highly Cross-Adsorbed Secondary Antibody, Alexa Fluor™ Plus 555	Thermo Fisher Scientific	Cat# A32732, RRID:AB_2633281
Goat anti-Mouse IgG (H+L) Highly Cross-Adsorbed Secondary Antibody, Alexa Fluor™ 488	Thermo Fisher Scientific	Cat# A-11029, RRID:AB_2534088
Goat anti-Mouse IgM (Heavy chain) Cross-Adsorbed Secondary Antibody, Alexa Fluor™ 555	Thermo Fisher Scientific	Cat# A-21426, RRID:AB_2535847
Donkey anti-Rat IgG (H+L) Highly Cross-Adsorbed Secondary Antibody, Alexa Fluor™ 488	Thermo Fisher Scientific	Cat# A-21208, RRID:AB_2535794
Goat anti-Guinea Pig IgG (H+L) Highly Cross-Adsorbed Secondary Antibody, Alexa Fluor™ 488	Thermo Fisher Scientific	Cat# A-11073, RRID:AB_2534117
Goat anti-Rabbit IgG (H+L) Secondary Antibody, HRP	Thermo Fisher Scientific	Cat# 31460 RRID:AB_228341
**Chemicals, peptides, and recombinant proteins**		

EZ-Link Sulfo-NHS-LC-Biotin	Thermo Fisher Scientific	A39257
EDTA-free protease inhibitors	Roche	1187358001
serine/threonine phosphatase inhibitors	Sigma Aldrich	P0044
tyrosine phosphatase inhibitors	Sigma Aldrich	P5726
BrdU (5-bromo-2'-deoxyuridine), Thymidine analog	Abcam	ab142567
Dulbecco's PBS w/o Calcium, w/o Magnesium	Euroclone	ECB4004L
Versene Solution	Thermo Fisher Scientific	15040066
Vitronectin (VTN-N) Recombinant Human Protein, Truncated	Thermo Fisher Scientific	A14700
RevitaCell™ Supplement (100X)	Thermo Fisher Scientific	A2644501
Essential 8™ Flex Medium Kit	Thermo Fisher Scientific	A2858501
Geltrex™ LDEV-Free, hESC-Qualified, Reduced Growth Factor Basement Membrane Matrix	Thermo Fisher Scientific	A1413302
Poli-L-ornitina	Sigma Aldrich	P3655-100MG
Laminin	Sigma Aldrich	L2020
DMEM/F-12, HEPES	Thermo Fisher Scientific	11330057
StemPro™ Accutase™ Cell Dissociation Reagent	Thermo Fisher Scientific	A1110501
STEMdiff™ SMADi Neural Induction Kit	STEMCELL Technologies	08581
STEMdiff™ Neural Progenitor Medium	STEMCELL Technologies	05833
Anti-Adherence Rinsing Solution	STEMCELL Technologies	07010
STEMdiff™ Neural Rosette Selection Reagent	STEMCELL Technologies	05832
Neurobasal™ Medium	Thermo Fisher Scientific	21103049
Neurobasal™ Plus Medium	Thermo Fisher Scientific	A3582901
B-27™ Supplement (50X), serum free	Thermo Fisher Scientific	17504044
B-27™ Plus Supplement (50X)	Thermo Fisher Scientific	A3582801
CultureOne™ Supplement (100X)	Thermo Fisher Scientific	A3320201
Human Recombinant BDNF	STEMCELL Technologies	78005
Human Recombinant GDNF	STEMCELL Technologies	78058
Retinoic acid	Sigma-Aldrich	R2625
L-Ascorbic acid 2-phosphate sesquimagnesium salt hydrate	Sigma-Aldrich	A8960
(-)-Bicuculline methochloride	abcam	ab120110
CNQX disodium salt	abcam	ab120044
D-AP5, NMDA glutamate site antagonist	abcam	ab120003
4x Laemmli Sample Buffer	Biorad	1610747
ProSieve™ QuadColor™ Protein Marker	Lonza	00193837
Neutral buffered formalin	Bio-Optica	05-01005Q
**Critical commercial assays**		
ECL Prime Western Blotting System	GE Healthcare	RPN2106
iScript™ cDNA Synthesis Kit	Biorad	1708891
SsoFast™ EvaGreen® Supermix	Biorad	1725201
RNeasy Mini Kit	Qiagen	74104
Chromium Next GEM Single Cell 3' Kit 4 reaz	10 Genomics	10X1000269
Chromium™ Next GEM Chip G Single cell kit 16 reaz	10 Genomics	10X1000127
NovaSeq 6000 S1 Reagent Kit v1.5	Illumina	20028319
Annexin V FITC Apoptosis Kit	Thermo Fisher Scientific	BMS500F1-100

**Deposited data**		
Single-cell RNA-seq data	This paper	GEO: GSE276494
**Experimental models: Cell lines**		
Control Human Induced Pluripotent Stem cells	Applied StemCell	ASE-9211
**Oligonucleotides (5’-3’)**		
SgRNA C1983A-g1	AAAGTAGACGACGTAGAAAA	
SgRNA C1983A-g2	AGAAAATGGACATCTCCATG	
C1983A ssODN g1&g2	CAAAGATATTGACAAAGAAGAAG GGGAACACCACAAAGTAGACGAC GTAGGAAATCGACATCTCCATGC GATACCCGGGGCTGGGGCCCTGG TTCTCAAAGGTGGCGTCCACCGA ATGCTT	
SgRNA C1983B-g1	CAGCGGTCGGATTCATTATA	
SgRNA C1983B-g2	TTTCTTCTCCTTTCAGCGGT	
C1983B ssODN g1&g2	GGACATTTCTTGCCTAAGCCGAG AGGGGGAGATATTACTCGTAATA AACTCTACATATCCTTATA GTGAATTCGACCGCTGAAAGGAG AAGAAAGGGGGTTAGTGCAGGCA ATGGGTTCACACGGGC	
**Primers for Sanger Sequencing (5’-3’)**		
hCav2.1 C1983A F	CCTGGGTGTTGTGTGTGTTT	
hCav2.1 C1983A R	CTGTCTCTCTCCTTCCTGCC	
hCav2.1 C1983B F	TCCATGGATGCTAGCAGGTT	
hCav2.1 C1983B R	CATCATGACCTCGCTGTGTG	
**Primers for Real Time PCR (5’-3’)**		
hCav2.1Total F	TGAATCTCTTTGTCGCCGTC	
hCav2.1Total R	ACACGCACGTACTCATCCA	
hCav2.1 EFa F	GTCCTCATAGGGTTGCTTGC	
hCav2.1 EFb F	CCTGGGTCTGGGGAAGAAGT	
hCav2.1 EFa/b R	GGCAGGTCCATCCGCAG	

Supplementary Figures

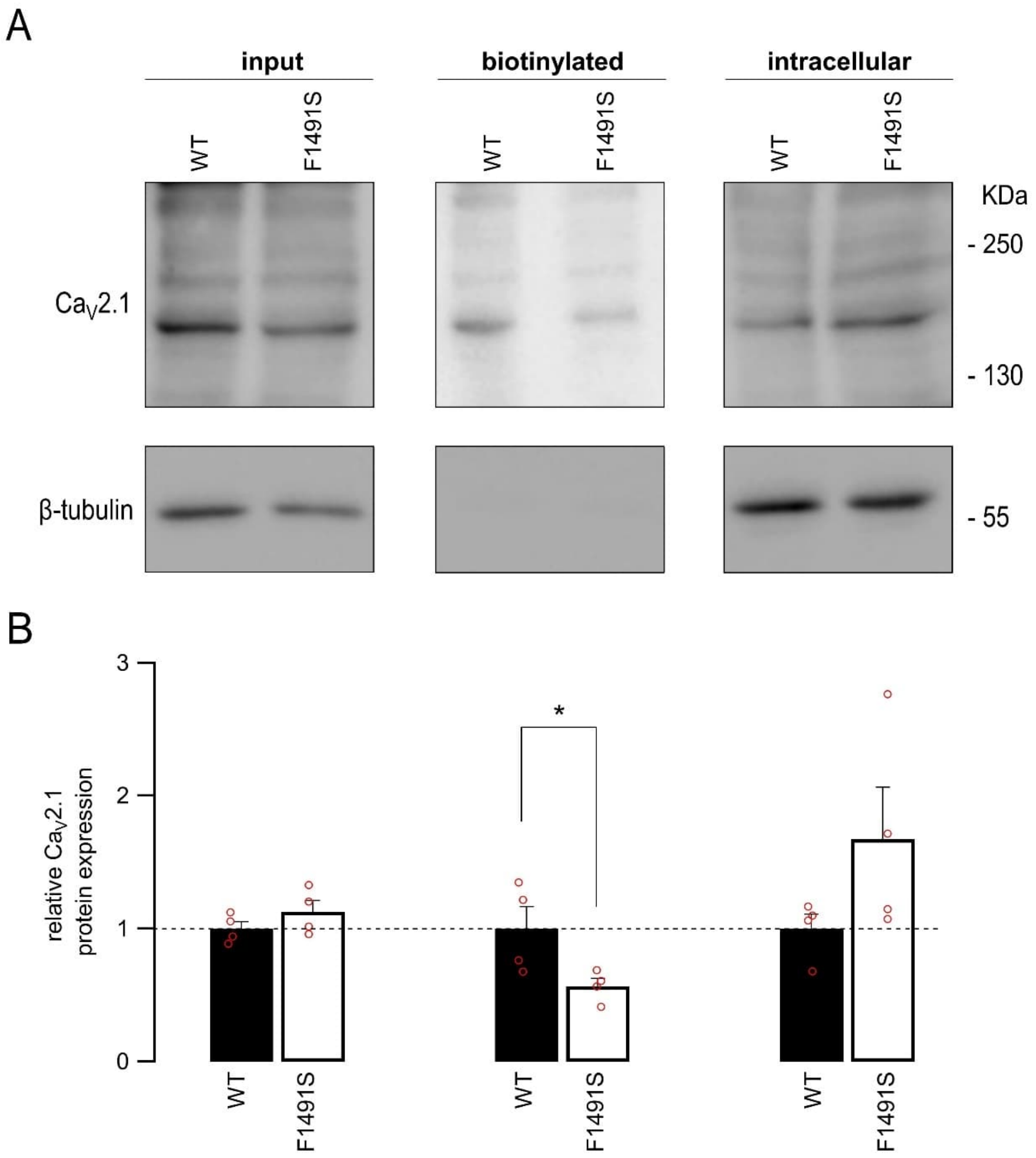

Fig. S1. The F1491S mutation reduces surface expression of $Ca_V2.1$. (A) Representative western blots of total (left, input), extracellular (middle, biotinylated) and intracellular (right) fractions from HEK293 cells expressing wild-type (WT) or F1491S $Ca_V2.1$ and the β2a and α2δ1auxiliary subunits. β-tubulin is used as a loading control. (B) Quantification of relative amount of WT and F1491S $Ca_V2.1$. Data are shown as mean ± SEM (bars) and single replicates (dots); *$p<0.05$, F1491S versus WT with the Student's t test (n=4).

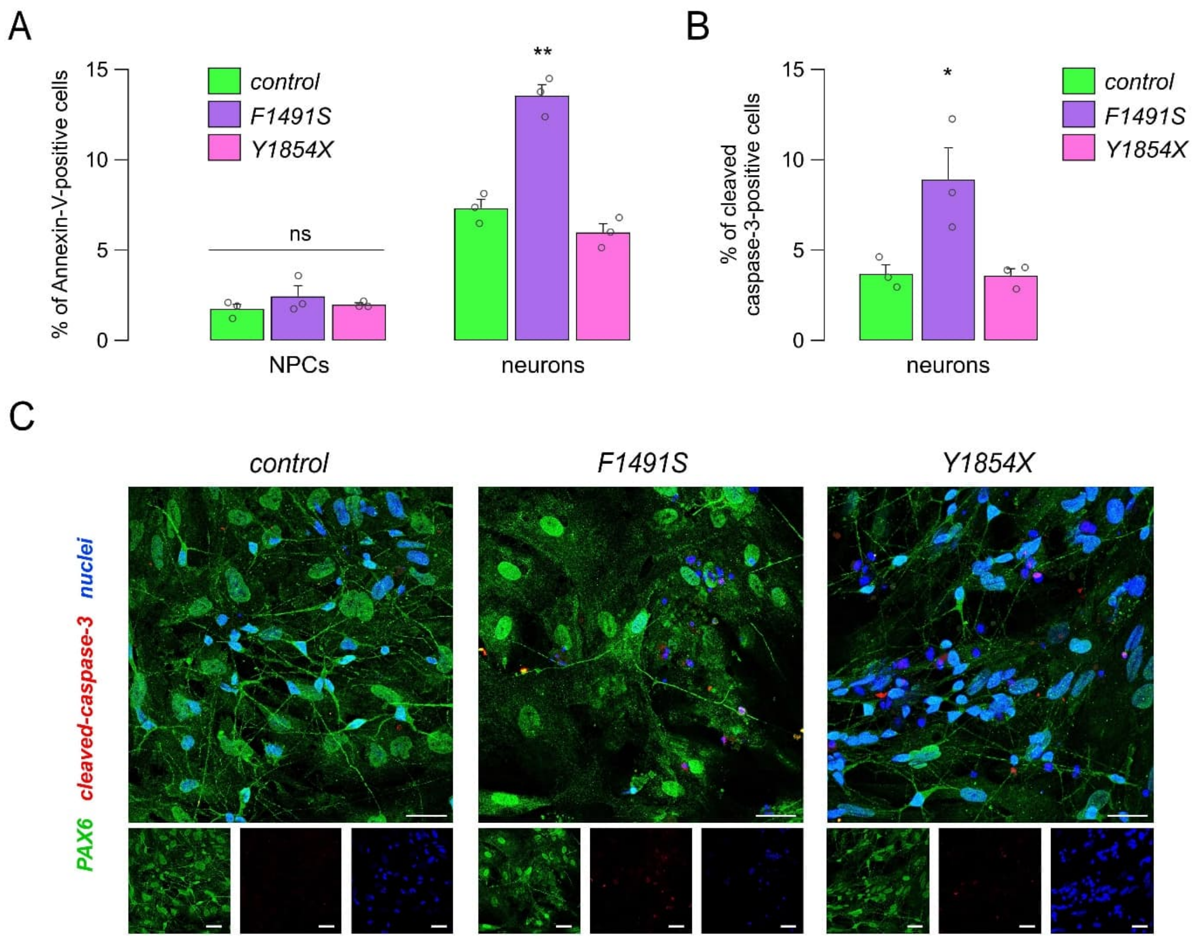

Fig. S2. Analysis of apoptosis in control and mutant cultures. (A) Apoptosis evaluation by Annexin-V and Propidium Iodide (PI) staining followed by flow cytometry analysis in control and mutated NPCs and neurons. (A) Graph showing the quantification of Annexin-V-positive / PI-negative cells. Data are shown as mean ± SEM (bars) and single replicates (dots); **p<0.01, F1491S versus WT with the Student's t test (n=3). (B) Apoptosis evaluation by immunofluorescence detection and quantification of cleaved-caspase-3 protein in control and mutated neurons. Data are shown as mean ± SEM (bars) and single replicates (dots); *p<0.05, F1491S versus WT with the Student's t test (n=3). (C) Representative immunofluorescence images of control and mutated neurons (10 DIV). Cells were labeled with antibodies directed against PAX6 and cleaved-caspase-3. Cells were also counterstained with DAPI to label cell nuclei. Scale bar: 20 μm.

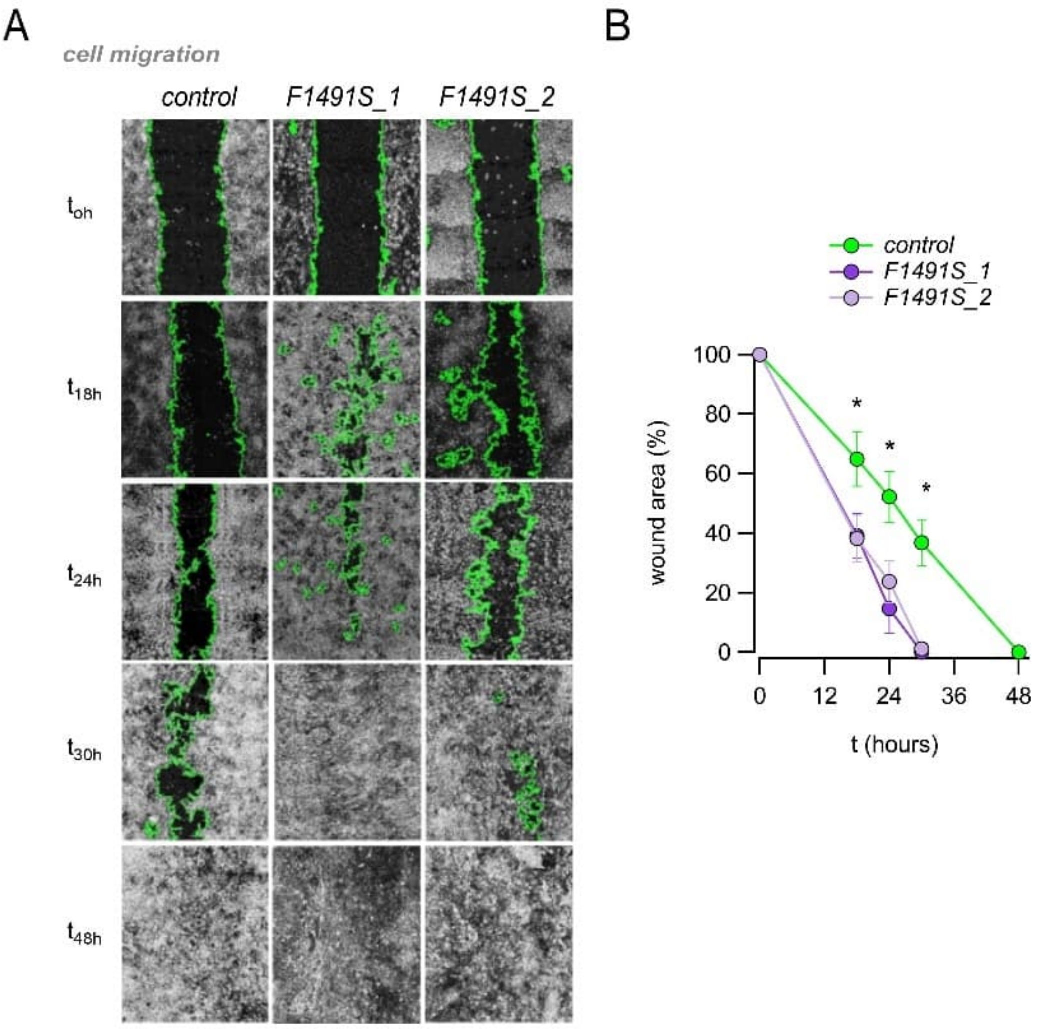

Fig. S3. *CACNA1A* loss-of-function caused by the F1491S mutation alters the migratory capacity of NPCs. Analysis of NPCs migration by wound healing assay in isogenic control and two independent clones carrying F1491S mutation (F1491S_1 and F1491S_2). Representative images (A) and analysis (B) of wounded areas of confluent neural progenitors at the indicated post-wounding time points. Wound edges, detected by image segmentation analysis with Image J, are outlined in green. Data are shown as mean ± SEM (n=3). * $p<0.05$ vs control (1-way ANOVA with Tukey's post hoc test).

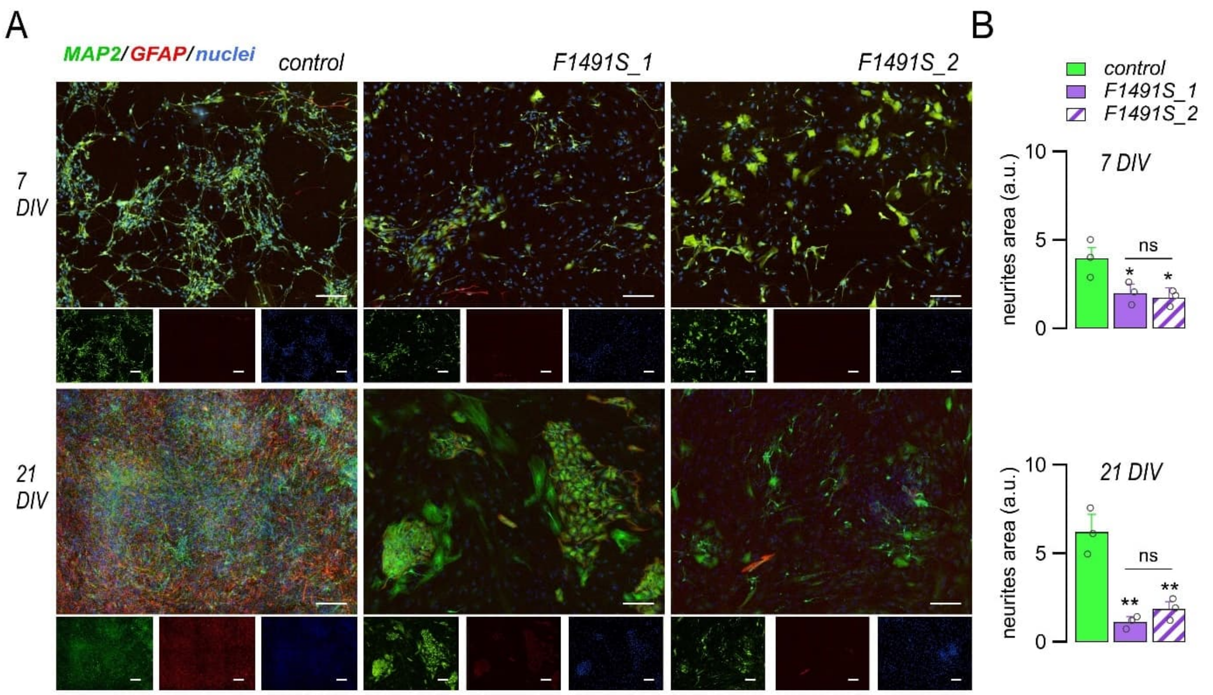

Fig. S4. ***CACNA1A*** **loss-of-function caused by F1491S mutation impairs neuronal generation.** Representative immunofluorescence images of control and mutated (two independent clones, F1491S_1 and F1491S_2) iPSC-derived neurons at 7 and 21 DIV. Cells were labeled with antibodies directed against MAP2 and GFAP as markers of neurons and glia, respectively. Cells were also counterstained with DAPI to label cell nuclei. Scale bar: 50 μm. (B) Graphs showing the quantification of neurite outgrowth. Data are shown as mean ± SEM (bars) and single replicates (dots), n=3. *, $p<0.05$; **, $p<0.01$ vs control (1-way ANOVA with Tukey's post hoc test).

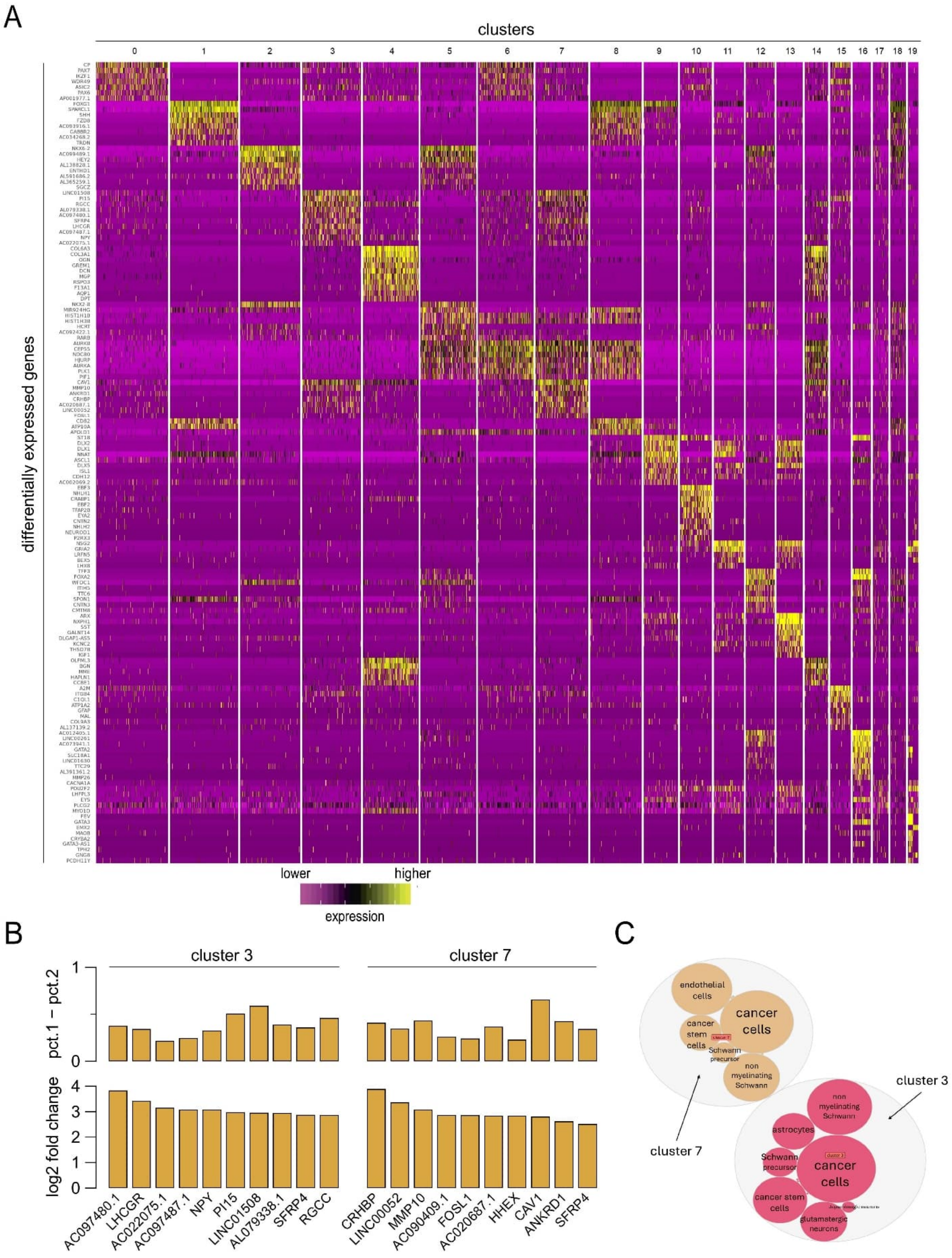

Fig. S5. Heterogenous gene expression in iPSC-derived NPCs. Heatmap showing the expression of marker genes for the indicated clusters obtained from scRNA-seq data of control and F1491S (F1491S_1) neural progenitors. The top 10 differentially expressed genes were selected and ranked based on cluster ID. Genes are shown in the left, cluster identifiers are shown on top; colored legend ranging from low (violet) to high

expression (yellow) is shown in the bottom. (B) Bar graphs reporting the differences in expression between pct.1 and pct.2 (top) (where pct.1 is the percentage of cells where the gene is detected in the specific cluster and pct.2 is the percentage of cells where the gene is detected on average in the other clusters) and the average fold changes (bottom) for the markers of clusters annotated as cancer cells (clusters 3 and 7). (C) Circle packing chart showing hierarchical data within clusters 3 and 7. The outer (grey) circles are relative to clusters, while the inner circles correspond to ScType cell types taken into consideration for cluster assignment. Circles size depends on the ScType scores. The biggest circle inside the grey circle corresponds to the cell type assigned to the entire cluster.

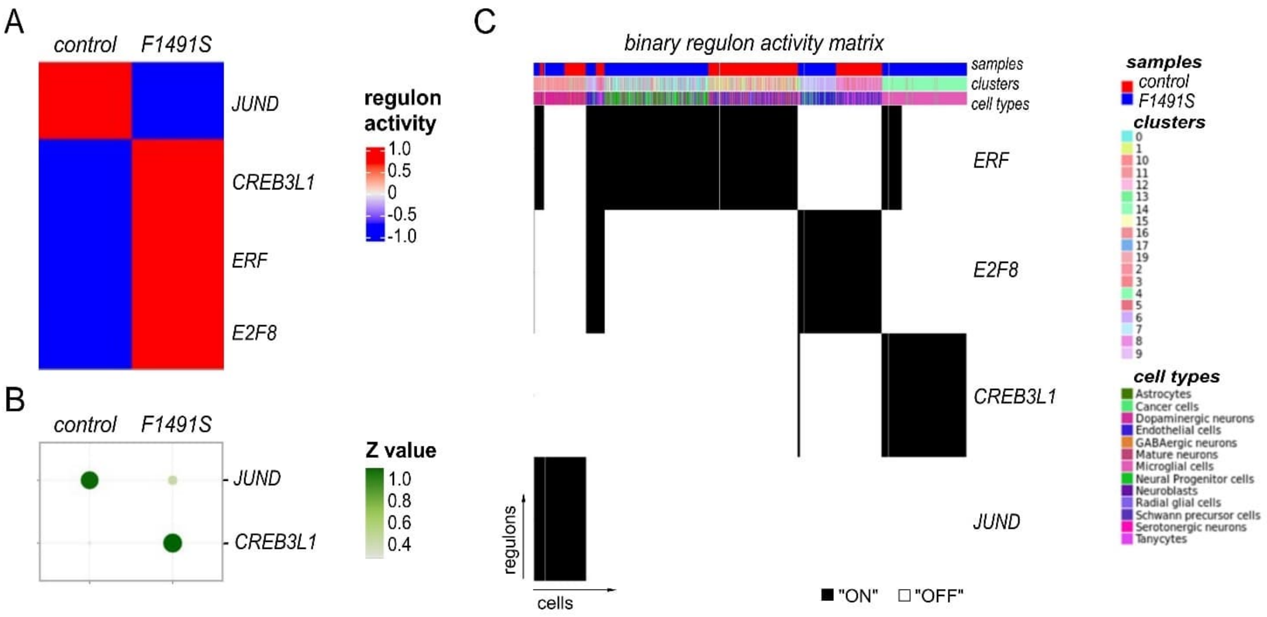

Fig. S6. NPCs carrying F1491S-CACNA1A show an altered transcriptional state. (A) Heatmap showing the regulon activity by sample. Regulon name is reported on the right. Sample names are reported on the top. (B) Dot plot showing regulons exceeding 0.01 of rss by samples. RSS value determines dot size while z value depicts dot color. (C) Binary activity matrix for regulons inferred by SCENIC: regulons were determined to be active ("ON", black) if they exceeded an automatically determined AUC regulon-specific threshold or inactive under this threshold ("OFF", white). After hierarchical clustering, clusters of regulons can be observed specifically for each cell population but also shared between the different cell populations.

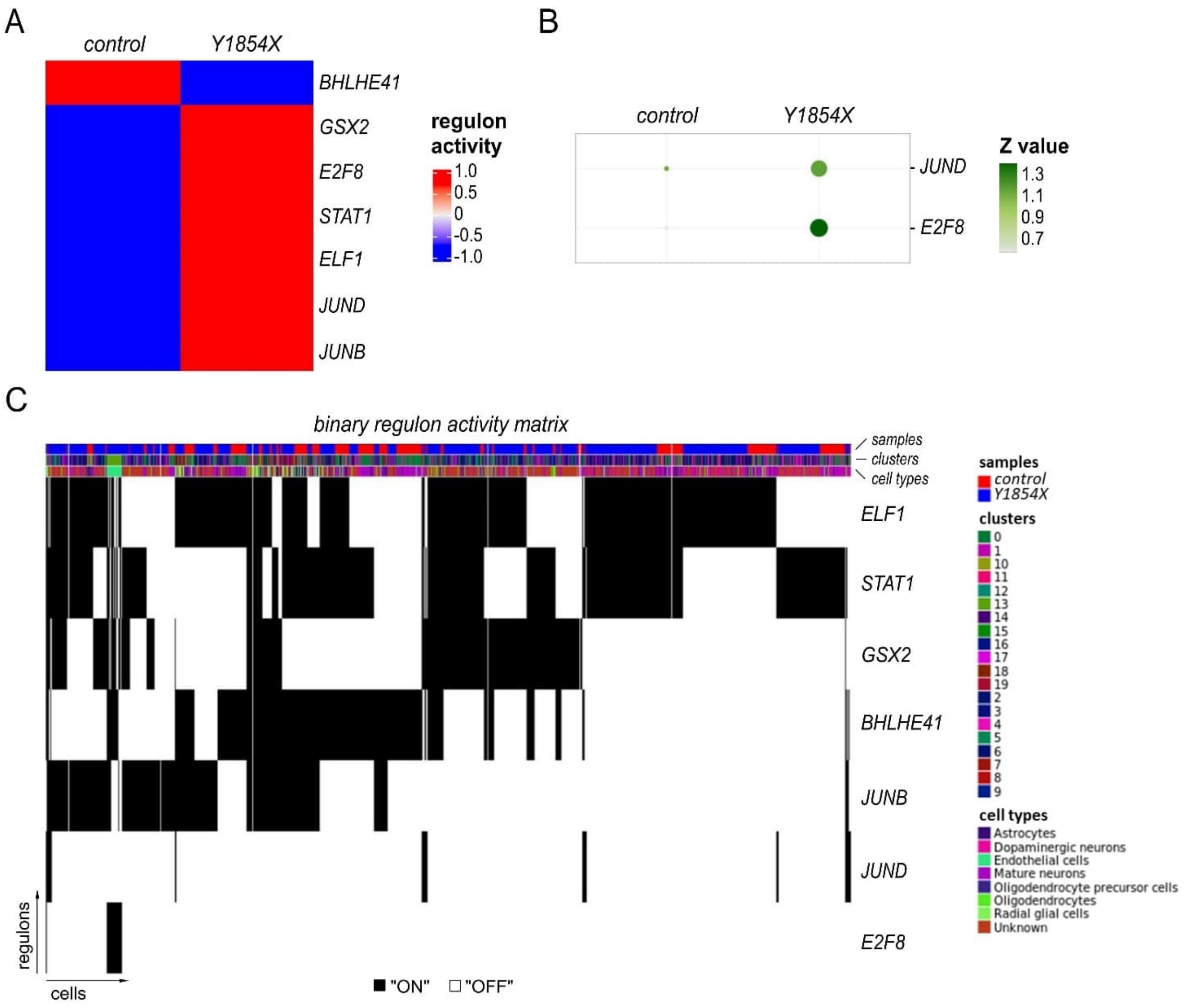

Fig. S7. iPSC-derived neurons carrying Y1854X-CACNA1A show an altered transcriptional state. (A) Heatmap showing the regulon activity by sample. Regulon name is reported on the right. Sample names are reported on the top. (B) Dot plot showing regulons exceeding 0.01 of rss by samples. RSS value determines dot size while z value depicts dot color. (C) Binary activity matrix for regulons inferred by SCENIC: regulons were determined to be active (“ON”, black) if they exceeded an automatically determined AUC regulon-specific threshold or inactive under this threshold (“OFF”, white).

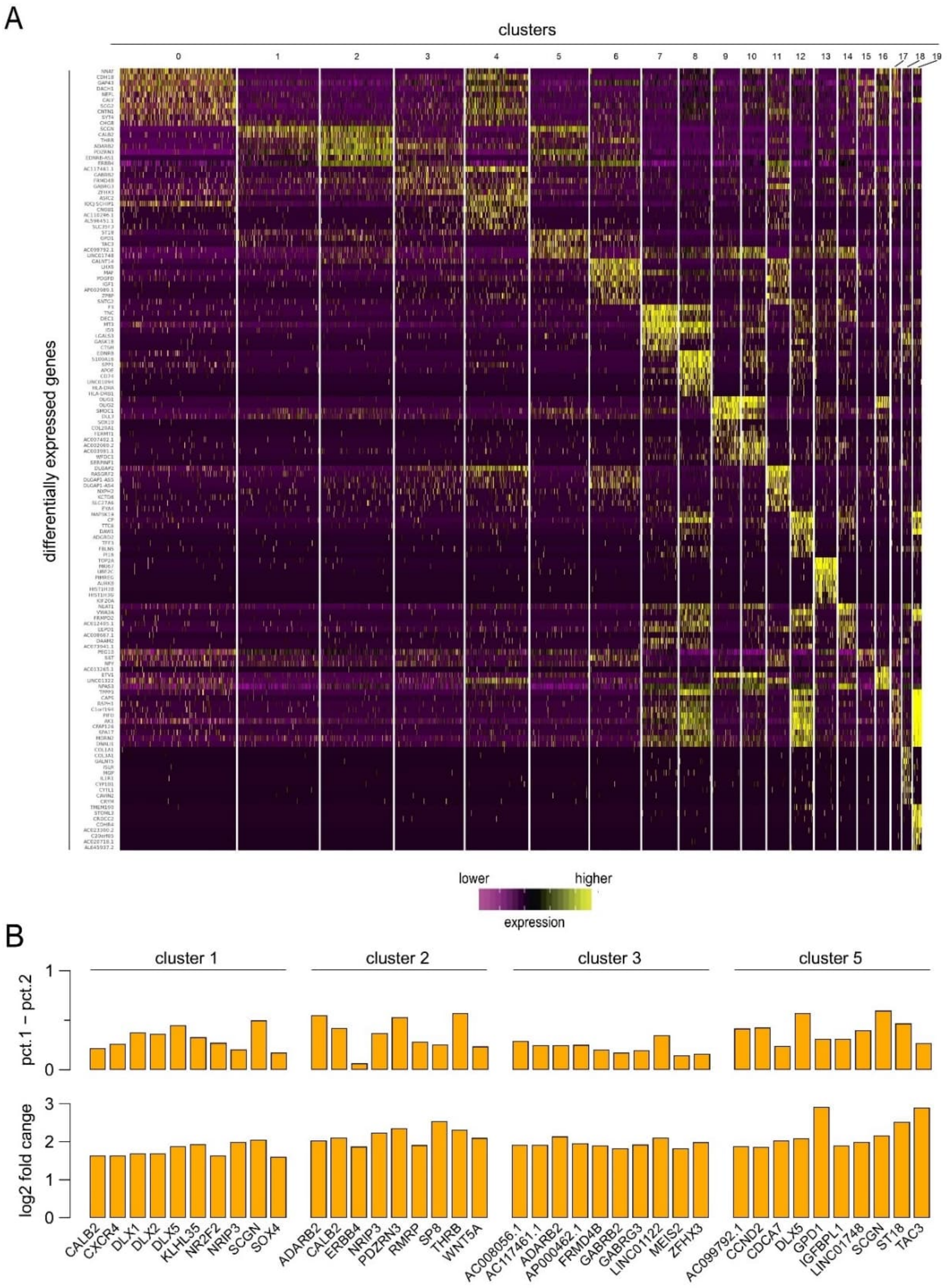

Fig. S8. Heterogenous gene expression in iPSC-derived neuronal cultures. Heatmap showing the expression of marker genes for the indicated clusters obtained from scRNA-seq data of control and Y1854X neurons at 49 DIV. The top 10 differentially expressed genes were selected and ranked based on cluster ID. Genes are shown in the left, cluster identifiers are shown on top; colored legend ranging from low (violet) to high expression (yellow) is shown in the bottom. (B) Bar graphs reporting the differences in expression between pct.1 and pct.2 (top) (where pct.1 is the percentage of cells where the gene is detected in the specific cluster and pct.2 is the percentage of cells where the gene is detected on average in the other clusters) and the average fold changes (bottom) for the markers of clusters annotated as unknown (clusters 1, 2, 3, and 5).

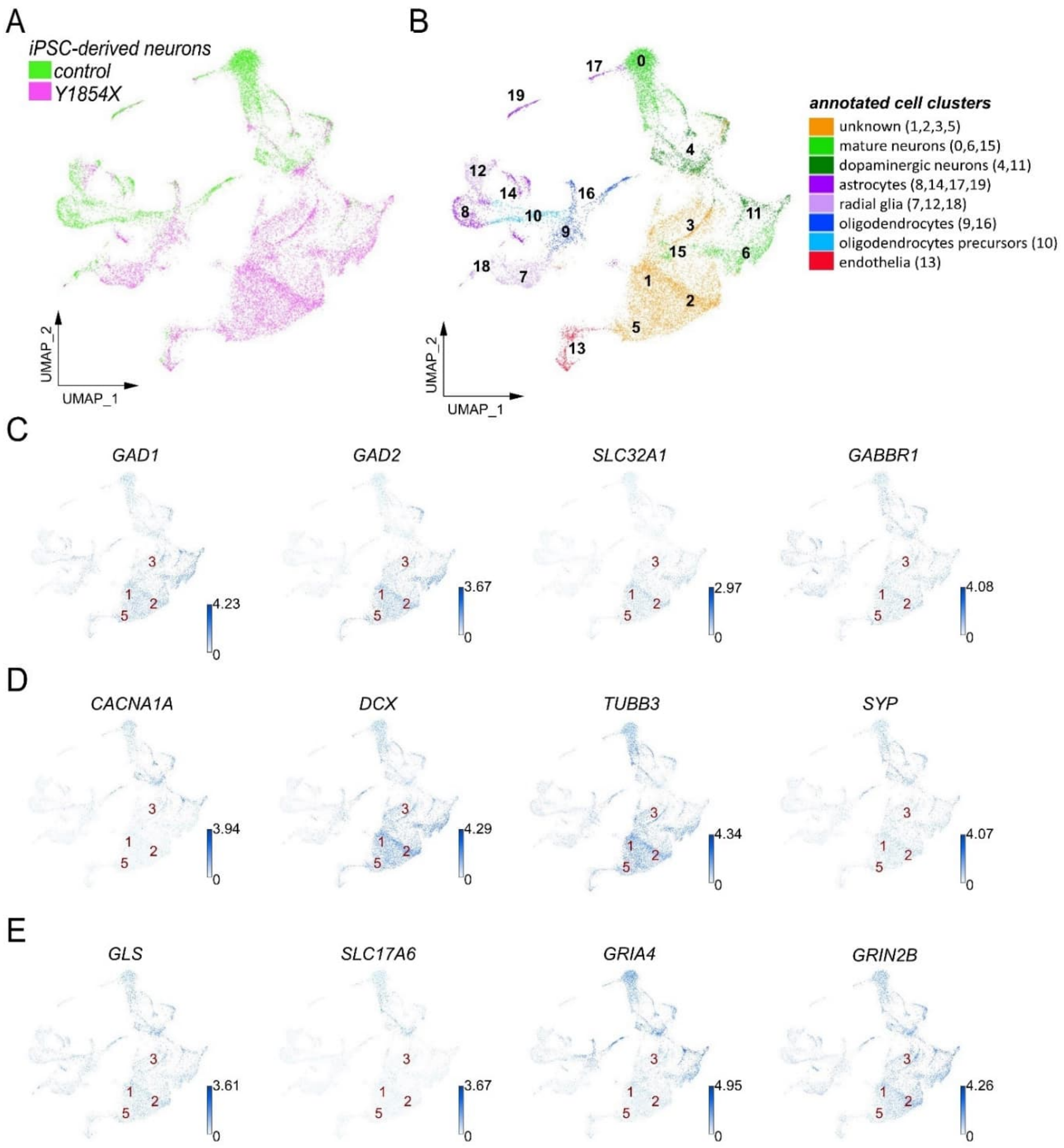

Fig. S9. *CACNA1A* loss-of-function caused by the Y1854X mutation alters excitatory-inhibitory balance of neural networks. (A, B) Global representation of gene expression through UMAP plot as also reported in Fig. 7. Each dot represents a single cell, whose position in the map reports the transcriptional similarity with respect to the neighbor cell. The different colors indicate the samples in (A), and the annotated ScType clusters in (B). (C) Feature plots showing the distribution and expression of the GABAergic markers *GAD1*, *GAD2*, *SLC32A1*, and *GABBR1*. Data are colored according to expression level. (D) Feature plots showing the distribution and expression of *CACNA1A*, and *DCX* and *TUBB3* as markers of immature neurons, and *SYP* as a marker of mature neurons. Data are colored according to expression level. (E) Feature plots showing the distribution and expression of the glutamatergic markers *GLS*, *SLC17A6*, *GRIA4*, and *GRIN2B*. Data are colored according to expression level.
